# Dietary Polyphenol-Based, Tumor Microenvironment-Responsive Carrier-Free Nanoplatforms for Synergistically Enhanced Tumor-Targeted Therapy

**DOI:** 10.3390/molecules31213895

**Published:** 2026-10-28

**Authors:** Liyan Yang, Xianqing Song, Zhonglei Wang

**Affiliations:** 1 School of Physics and Physical Engineering, Qufu Normal University, Qufu 273165, China; 2 Beijing National Laboratory for Molecular Sciences, Institute of Chemistry, Chinese Academy of Sciences, Beijing 100045, China; 3 Center for Nanomedicine, Key Laboratory of Green Natural Products and Pharmaceutical Intermediates in Colleges and Universities of Shandong Province, School of Chemistry and Chemical Engineering, Qufu Normal University, Qufu 273165, China; 4 Affiliated Hospital Group of Guangdong Medical University Shenzhen Baoan Central Hospital (Baoan Central Hospital of Shenzhen), Shenzhen 516003, China; 5 Key Laboratory of Bioorganic Phosphorus, Chemistry & Chemical Biology (Ministry of Education), School of Pharmaceutical Sciences, Tsinghua University, Beijing 100084, China

**Keywords:** dietary polyphenol, carrier-free, smart nanoplatforms, antitumor, batch-to-batch

## Abstract

Cancer remains a leading global cause of morbidity and mortality. Natural polyphenols show potent antitumor activity—driven by antioxidant, anti-angiogenic, and epigenetic regulatory effects—but their clinical use is hindered by poor aqueous solubility, chemical instability in physiological conditions, low oral bioavailability, and insufficient tumor accumulation. In response, carrier-free nanomedicines, self-assembled nanostructures formed exclusively from therapeutic polyphenols without exogenous excipients or polymeric carriers, have emerged as a paradigm-shifting delivery strategy. This review provides a rigorous, mechanism-informed evaluation of how dietary polyphenol (e.g., epigallocatechin gallate [EGCG], curcumin, resveratrol, mangiferin, and quercetin)-based carrier-free smart nanoplatforms, by virtue of their tunable supramolecular architecture, enhanced stability, prolonged circulation, and passive/active tumor targeting capabilities, strategically exploit and amplify these antitumor mechanisms to confront the heterogeneity, adaptability, and therapeutic resistance inherent in malignant pathophysiology. Crucially, this review bridges translational gaps by explicitly linking molecular-level mechanistic insights to rational nanomaterial design principles and quantifiable preclinical/clinical therapeutic outcomes. Finally, it critically appraises persistent scientific, manufacturing, and regulatory challenges, including scalability, batch-to-batch reproducibility, long-term colloidal stability, in vivo fate mouthline standardized efficacy/safety assessment, and outlines forward-looking perspectives for accelerating the clinical realization of polyphenol-based carrier-free smart nanotherapeutics.

## 1. Introduction

Cancer is a complex, systemic, and inherently invasive disease characterized by uncontrolled cellular proliferation, evasion of growth suppression, resistance to cell death, replicative immortality, induction of angiogenesis, activation of invasion and metastasis, dysregulation of cellular energetics, and avoidance of immune destruction—hallmarks collectively underpinned by progressive, cumulative genomic and epigenomic alterations [1,2,3,4,5]. Thus, cancer must be understood not merely as a localized neoplasm but as a dynamic, evolving, and systemic disorder rooted in decades-long molecular pathogenesis [6,7]. Although substantial advancements have been achieved in diagnostic modalities, including high-resolution imaging, liquid biopsy, and molecular profiling, as well as in surgical techniques (e.g., minimally invasive and robotic-assisted procedures), precision radiotherapy (e.g., stereotactic body radiotherapy and proton beam therapy), pharmacotherapy (encompassing targeted agents, antibody–drug conjugates, and epigenetic modulators), and immunotherapy (notably immune checkpoint inhibitors, bispecific T-cell engagers, and chimeric antigen receptor T-cell therapies), oncological management continues to encounter severe, multifaceted hurdles [8,9,10,11,12,13,14,15].

These persistent physiological, pharmacokinetic, and target-related obstacles (such as off-target binding, nonspecific tissue accumulation, immune-mediated cytotoxicity, and on-target/off-tumor effects) drive sustained, multidisciplinary research efforts aimed at enhancing therapeutic specificity while simultaneously minimizing secondary injury to healthy tissues [16,17]. Such endeavors encompass rational drug design strategies, including structure–activity relationship (SAR) optimization, prodrug engineering, and stimuli-responsive release systems, as well as advanced delivery platforms like antibody–drug conjugates, nanocarriers with ligand-mediated targeting, and cell-based therapeutics engineered for spatially restricted activation [18,19,20,21,22,23,24,25]. Crucially, these innovations are pursued without compromising intrinsic pharmacological potency; rather, they seek to decouple efficacy from toxicity by improving the therapeutic index through precision targeting, controlled biodistribution, and temporal modulation of drug action. Collectively, these coordinated scientific and clinical initiatives reflect a paradigm shift from empirically optimized dosing toward mechanism-informed, patient-tailored interventions, where enhanced specificity and reduced collateral damage are not merely desirable endpoints but foundational requirements for next-generation therapeutics.

**Figure 1:**
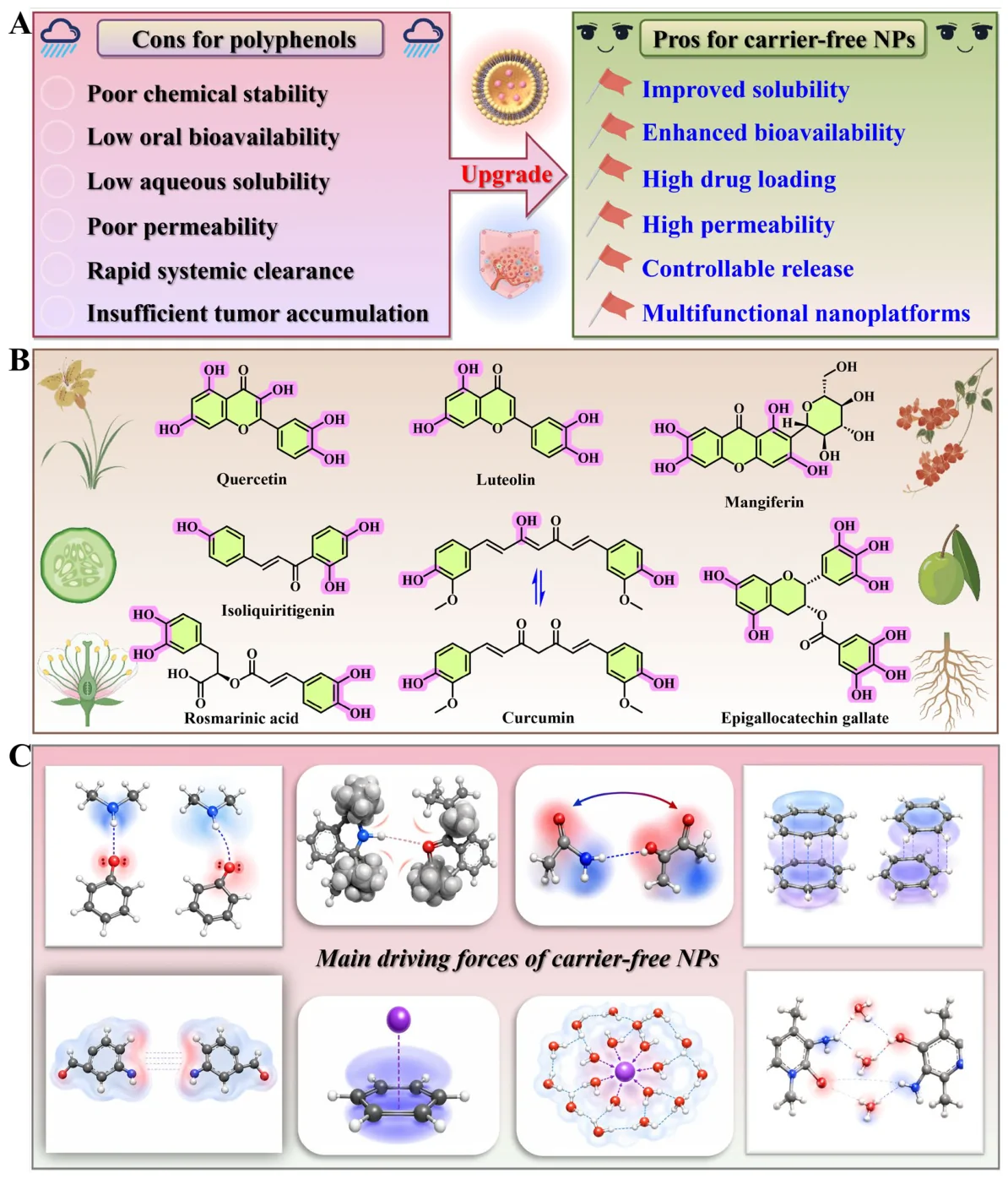
(**A**) Advantages of carrier-free nanoparticles (NPs) in overcoming the limitations of polyphenols. (**B**) Chemical structures of natural polyphenols. (**C**) Main driving forces of carrier-free NPs.

Natural products have long occupied a pivotal position in drug discovery and development, attracting sustained and intensive interest from medicinal chemists, pharmacologists, and translational researchers [26,27,28,29,30,31,32,33,34,35]. This enduring fascination stems primarily from their extraordinary structural diversity, encompassing complex polyphenols, terpenoids, alkaloids, glycosides, and macrolides, many of which exhibit potent, selective, and mechanistically distinct anticancer activities [36,37,38,39,40,41,42,43,44,45]. Unlike synthetic compounds, natural products often possess privileged scaffolds evolved through millions of years of ecological interaction, conferring favorable pharmacokinetic properties, target promiscuity with therapeutic relevance, and enhanced ability to modulate challenging biological targets such as protein–protein interactions or undruggable transcription factors. Numerous clinically approved anticancer agents, including paclitaxel, doxorubicin, and camptothecin derivatives, are either natural products or direct semi-synthetic analogs, underscoring their irreplaceable contribution to modern cancer therapeutics. Contemporary advances in metabolomics, genome mining, heterologous biosynthesis, and AI-driven natural product annotation are further accelerating the identification and optimization of novel anticancer leads. Consequently, natural products remain not merely historical cornerstones but dynamic, integrative platforms for next-generation oncology innovation, bridging chemical biology, systems pharmacology, and precision medicine paradigms. Naturally active polyphenols represent a compelling class of phytochemicals with well-documented, robust, and multimodal antitumor activity. Their therapeutic efficacy is underpinned by a constellation of well-characterized pharmacological properties, most notably potent antioxidant capacity, inhibition of pathological angiogenesis, and modulation of epigenetic machinery. Collectively, these mechanisms enable polyphenols to simultaneously target multiple oncogenic hallmarks, including sustained proliferative signaling, evasion of growth suppressors, resistance to cell death, tumor-promoting inflammation, and dysregulated cellular metabolism. Despite this exceptional mechanistic breadth and favorable safety profile in preclinical models, the clinical translation of polyphenols has been persistently hampered by profound intrinsic limitations rooted in their suboptimal physicochemical and pharmacokinetic behavior (Figure 1A). Specifically, many bioactive polyphenols (Figure 1B), such as epigallocatechin gallate (EGCG), curcumin, resveratrol, mangiferin, and quercetin, exhibit marked chemical lability under physiological pH and enzymatic conditions; suffer from low oral bioavailability (<1–5% in most cases) due to poor aqueous solubility, limited membrane permeability, and extensive phase II conjugation coupled with rapid hepatic first-pass metabolism; and demonstrate insufficient tumor-selective accumulation owing to nonspecific systemic distribution, and short plasma half-lives.

In response to these persistent delivery bottlenecks, polyphenol-based carrier-free smart nanoplatforms, defined as self-assembled, therapeutically active nanostructures composed exclusively of polyphenolic molecules without any exogenous excipients, synthetic polymers, lipids, or inorganic scaffolds, have emerged as a paradigm-shifting strategy in nanotherapeutics (Figure 1A). Unlike conventional nanoparticle formulations that rely on inert carriers to encapsulate or conjugate drugs, carrier-free systems leverage the intrinsic amphiphilicity, π–π stacking propensity, hydrogen-bonding capacity, and metal-chelating functionality of polyphenols to drive spontaneous supramolecular assembly into stable, nanoscale architectures (Figure 1C) [46,47,48,49,50]. This approach preserves 100% drug payload, eliminates carrier-related toxicity and immunogenicity concerns, and enables precise structure–function tuning through rational molecular engineering.

The introduction of the “carrier-free” concept marks a significant paradigm shift in the design philosophy of nanomedicines. “Carrier-free” emphasizes that drug molecules self-assemble into nanoscale aggregates through structural design and intermolecular forces, without relying on external carrier materials for encapsulation or loading. In the academic literature, terms such as “carrier-free nanomedicines” and “carrier-free nanodrugs” are also used. The theoretical necessity and feasibility of high drug loading design represent another core pillar of the carrier-free strategy. From a therapeutic standpoint, many anticancer drugs have narrow therapeutic windows, requiring high local drug concentrations to achieve effective cytotoxicity. Traditional carrier systems, however, are often limited by the carrier-to-drug ratio, making it difficult to meet this requirement. From a pharmaceutical perspective, high drug loading enables reduced dosing volume and frequency, thereby improving patient compliance and quality of life. The carrier-free approach achieves this through rational design of prodrug molecules, endowing the drug itself with amphiphilicity or multiple intermolecular interaction capabilities, allowing spontaneous self-assembly into nanostructures in aqueous environments. The thermodynamic basis of this strategy lies in the hydrophobic collapse of the hydrophobic drug core, driven by hydrophobic effects, which releases the binding free energy of ordered water layers and forms a thermodynamically favorable aggregated state. Kinetically, moderate intermolecular interaction strengths and flexible molecular conformations facilitate reversible control of the assembly process and dynamic stability of the final nanostructure. “Carrier-free nanoplatforms”, “pure drug nanoparticles”, and “carrier-free nanoassemblies” are often used interchangeably to describe this emerging field. The theoretical advantage of the carrier-free strategy in eliminating carrier-related toxicity is grounded in a thorough analysis of underlying biological mechanisms (Table 1). Although polymer-based nanocarriers (such as PEG, PLGA, and HPMA copolymers) have been FDA-approved for specific indications, their long-term fate in vivo remains uncertain. Some polymeric carriers, due to their high molecular weight or highly stable structures, are difficult to effectively metabolize and eliminate, potentially leading to accumulation and toxicity in reticuloendothelial system organs such as the liver and spleen. Moreover, the carrier materials themselves may activate the complement system, induce antibody production, or trigger allergic reactions, resulting in what is known as “nanocarrier-induced toxicity”. By eliminating exogenous carrier materials, the carrier-free approach fundamentally avoids these risks, allowing safety evaluation of drug delivery systems to return to the intrinsic toxicological profile of the drug molecule itself, thereby significantly simplifying the complexity of safety assessment. Additionally, removing carrier components reduces the immunogenicity risk of nanomedicines and minimizes unexpected pharmacokinetic behaviors caused by carrier-drug interactions.

The theoretical necessity and feasibility of high drug loading design represent another core pillar of the carrier-free strategy (Table 1). From a therapeutic standpoint, many anticancer drugs have narrow therapeutic windows, requiring high local drug concentrations to achieve effective cytotoxicity. Traditional carrier systems, however, are often limited by the carrier-to-drug ratio, making it difficult to meet this requirement. From a pharmaceutical perspective, high drug loading enables reduced dosing volume and frequency, thereby improving patient compliance and quality of life. The carrier-free approach achieves this through rational design of drug molecules, endowing the drug itself with amphiphilicity or multiple intermolecular interaction capabilities, allowing spontaneous self-assembly into nanostructures in aqueous environments. The thermodynamic basis of this strategy lies in the hydrophobic collapse of the hydrophobic drug core, driven by hydrophobic effects, which releases the binding free energy of ordered water layers and forms a thermodynamically favorable aggregated state. Kinetically, moderate intermolecular interaction strengths and flexible molecular conformations facilitate reversible control of the assembly process and dynamic stability of the final nanostructure. It is noteworthy that drug loading capacity and encapsulation efficiency are two distinct parameters; however, they exhibit an intrinsic interrelationship. Under conditions of a fixed total amount of drug input, enhancing the encapsulation efficiency—i.e., encapsulating a greater proportion of the drug—typically leads to an increase in drug loading capacity, provided that the total mass of the nanoparticles does not increase significantly. Conversely, if the encapsulation efficiency is improved by increasing the amount of co-assembling drugs, the resulting increase in NP mass may paradoxically lead to a decrease in drug loading capacity.

The carrier-free strategy demonstrates significant economic advantages in simplifying preparation processes, improving batch consistency, and reducing production costs. Traditional nanomedicine formulations involve multiple steps, including synthesis and purification of carrier materials, drug encapsulation, and complex post-processing purification, each of which may introduce batch-to-batch variability. In contrast, the carrier-free approach integrates the formation of nanostructures with the chemical synthesis of drug molecules, enabling the direct production of target nanomedicines through one-step or simplified assembly processes, thereby greatly shortening the manufacturing timeline. Enhanced batch consistency arises from fewer quality control checkpoints and the direct correlation between critical quality attributes and molecular structure: if the chemical structure and purity of the prodrug molecule are maintained, its self-assembly behavior becomes highly predictable and reproducible. From an industrial perspective, process simplification translates into lower equipment investment, shorter production cycles, and higher manufacturing efficiency, all of which are crucial for reducing the cost and enhancing clinical accessibility.

Carrier-free nanoplatforms have emerged as a prominent research focus in the field of nanoscale drug delivery in recent years. Their defining feature lies in the deliberate elimination of conventional polymeric or inorganic carriers; instead, therapeutic agents—or other bioactive components—self-assemble into nanostructures via non-covalent interactions, thereby enabling high drug loading, minimized carrier-associated toxicity, and full traceability of constituent components. However, the term “carrier-free” has, in practice, significantly diverged from its original strict definition. This conceptual expansion is evident in multiple scenarios: when metal ions are incorporated into drug self-assembly systems to enhance structural stability or confer chemodynamic therapeutic functionality; when targeting ligands are covalently conjugated to drug molecules to impart active targeting capability; and when polyphenolic molecules are employed to surface-modify drug-based nanoparticles to improve colloidal stability. Collectively, these strategies continue to broaden the functional scope and application boundaries of “carrier-free” nanoplatforms.

**Figure 2:**
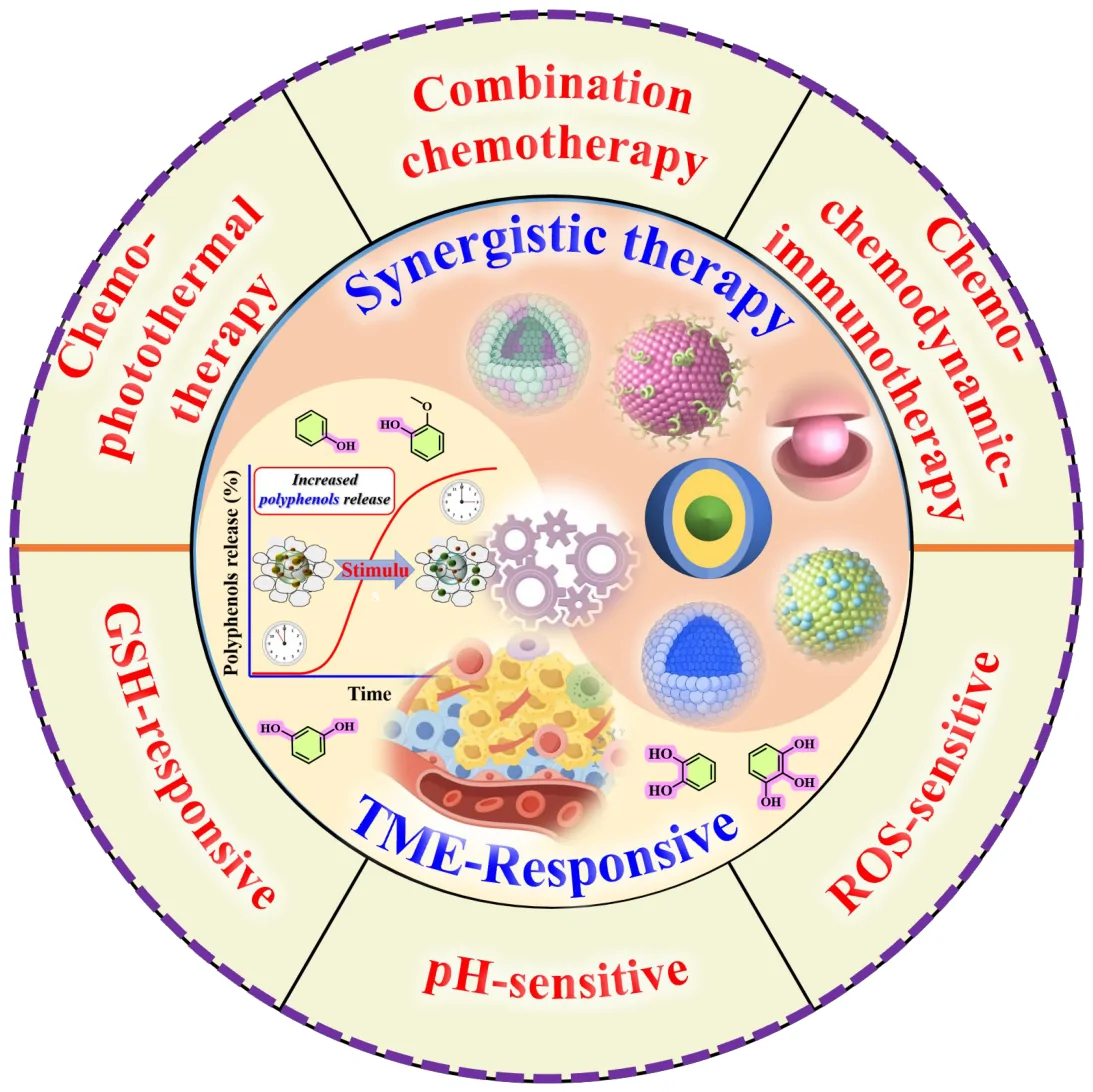
Polyphenol-based, TME-responsive, carrier-free nanoplatforms for synergistic tumor therapy.

This review delivers a rigorous, mechanism-informed synthesis of how polyphenol-based carrier-free smart nanoplatforms strategically exploit and synergistically amplify native antitumor pathways (Figure 2, Table 2). Critically, we systematically correlate nanoplatform attributes (size, surface charge, assembly kinetics, disassembly triggers) with quantifiable preclinical outcomes (tumor growth inhibition, metastasis suppression, immune cell infiltration). Finally, the review undertakes a balanced, critical appraisal of enduring scientific, manufacturing, and regulatory challenges impeding clinical advancement. We conclude with forward-looking perspectives to accelerate the rational development and regulatory approval of next-generation polyphenol-based carrier-free smart nanotherapeutics.

## 2. Flavonoid-Based Carrier-Free Nanoplatform

A flavonoid-based carrier-free nanoplatform represents an emerging paradigm in nanomedicine that synergistically integrates the intrinsic pharmacological properties of natural flavonoids with advanced nanoscale engineering principles. Structurally, the platform exploits the amphiphilicity, π–π stacking propensity, and hydrogen-bonding capacity inherent to flavonoid scaffolds (e.g., quercetin, epigallocatechin gallate, luteolin, or fisetin) to drive spontaneous supramolecular organization into well-defined nanoparticles (typically 20–150 nm in hydrodynamic diameter) under mild aqueous conditions. These nano assemblies retain high drug loading efficiency (theoretically approaching 100% *w*/*w*), exceptional colloidal stability across physiological pH and ionic strength ranges, and favorable pharmacokinetic behavior, including prolonged systemic circulation, enhanced tumor accumulation via the enhanced permeability and retention (EPR) effect, and efficient cellular internalization mediated by flavonoid-specific membrane interactions.

### 2.1. Isoliquiritigenin-Based Nanoplatform for Tumor Chemo-Chemodynamic-Immunotherapy

TME features hypoxia, relatively acidic pH, high ROS and GSH levels, and immune dysregulation, distinguishing tumor tissue from normal tissue and driving therapy resistance [68]. Transition metal (Fe, Mn, Cu, Co) NPs can respond to the TME by consuming endogenous H_2_O_2_ and triggering Fenton/Fenton-like reactions to generate abundant •OH, enabling CDT [69,70]. However, a single CDT is not always sufficient, and it may also require a high dose with toxicity; researchers combine CDT with chemotherapy [71] and immunotherapy [72] to strengthen the antitumor effect but reduce the side effects. Isoliquiritigenin (ISL) is a chalcone extracted from *Glycyrrhiza* roots (*G. uralensis*, *G. inflata*, *G. glabra*, etc.), used in food additives and disease therapy that possesses strong anticancer [73], anti-inflammatory [74], and antioxidant activities [75] through induction of apoptosis, cell cycle arrest, and inhibition of angiogenesis. However, ISL is hydrophobic and has limited solubility, which poses complications when addressing delivery, efficacy, and clinical translation [76,77]. *Rosa laevigata* polysaccharide (RLP) has innate antioxidants [78], immunomodulatory [79], and antitumor [80] effects, as well as good biocompatibility and degradation [81], which makes it a potential nanocarrier material. Loading ISL onto this RLP-based nanocarrier could improve its solubility and bioavailability and facilitate targeted delivery.

**Figure 3:**
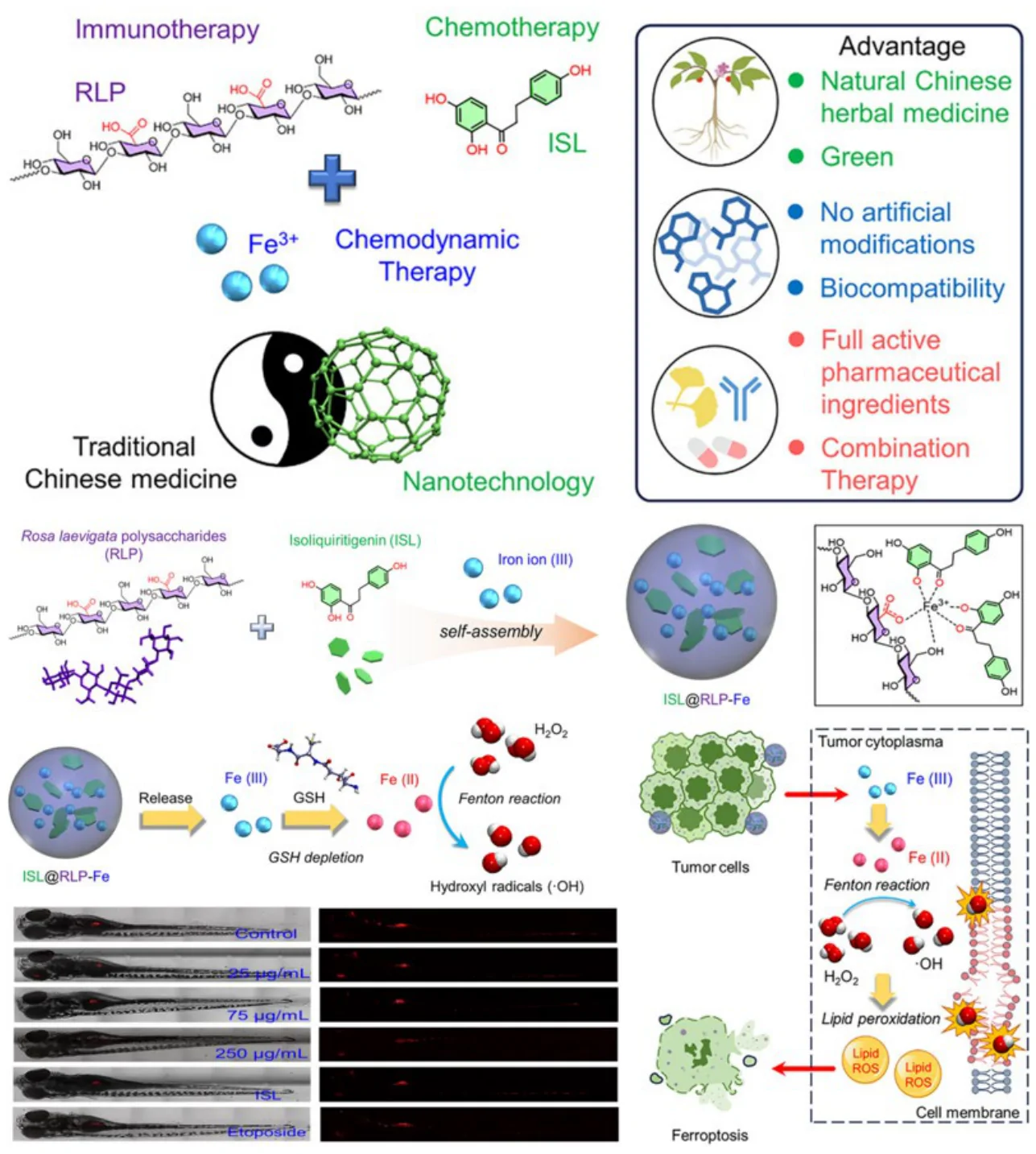
Schematic of carrier-free multifunctional nanoparticle system ISL@RLP-Fe for synergistic tumor chemo-chemodynamic-immunotherapy. Reproduced from Ref. (Lin et al., 2025) [54] with permission from © 2025 Wiley-VCH GmbH.

Lin et al. [54] used natural product self-assembly to create a fully active API nano-system (ISL@RLP-Fe) where all components co-assembled without carriers, markedly boosting loading, biocompatibility, and safety (Figure 3). ISL is a chemotherapeutic drug, whereas RLP provides a structural framework and immunostimulatory function. Fe^3+^ acts as a catalyst for CDT by simultaneously coordinating with the ISL phenolic hydroxyl and ketone carbonyl groups and with RLP’s carboxyl groups of RLP, driving the spontaneous assembly of the RLP–ISL nano-metal chelate. Dynamic light scattering yields a hydrodynamic diameter of 120.9 nm (PDI = 0.210); transmission electron microscopy shows ~85 nm with uniform morphology; zeta potential is approximately −18.4 mV. Loading content and loading efficiency are: ISL DLC 4.0% (DLE 48.5%), RLP DLC 92.9% (DLE 69.7%), Fe^3+^ DLC/DLE 89.8%. ISL@RLP-Fe remained stable in particle size and PDI over 20 days at 25 °C and showed stability in PBS and physiological saline. ISL@RLP-Fe showed a TME-responsive, three-synergistic antitumor effect.

Stabilized at pH 7.4, it releases ISL slowly ~18%/24 h. Under pH 6.0, the nano assembly swells, rapidly releasing ISL to ~91% at 24 h. Under dual stimulus of pH 6.0 and 10 mM GSH, the assembly quickly disassembles within 1 h releasing ~60% ISL. It releases nearly all at 8 h under the driving forces of carboxyl protonation and competitive Fe^3+^/GSH coordination with ISL. At the in vitro cellular level, ISL@RLP-Fe demonstrated significant dose-dependent cytotoxicity towards both MCF-7 and HepG2 cells, with IC_50_ values of 17.4 μg/mL and 19.0 μg/mL, respectively. This effect was significantly better than that of free ISL at the same concentration. The in vivo effects were evaluated using a zebrafish MCF-7 xenograft tumor model. The results showed that compared with the control group, ISL@RLP-Fe (25, 75, 250 μg/mL) could significantly reduce tumor volume (inhibition rates were 14.9%, 57.5%, and 77.7%, respectively) and inhibited the migration and spread of cancer cells (reduction rates of metastatic foci were 14.2%, 50.8%, and 74.1%, respectively). This effect was significantly better than that of free ISL at the same concentration. This study demonstrated its in vivo synergistic antitumor activity. Mechanistically, released ISL exerted its cytotoxic and apoptosis induction effect through direct inhibition of tumor cell proliferation through the PI3K/Akt and NF-κB pathways, and exhibited dose-dependent cytotoxic effects on both MCF-7 and HepG2 cells compared to that of free ISL, accompanied by ROS generation. The significant decrease in tumor cell viability indicated that elevated levels of GSH reduced ferrous Fe^3+^ to Fe^2+^, which led to a Fenton reaction. This produced hydroxyl radicals, •OH, lipid peroxidation, and ferroptosis induction. RLP acted as a stable nano-frame and immune booster, triggering dendritic cells and significantly increasing CD40 (16.6–76.1-fold) and CD80, CD86, MHC-II, for enhancing the antigen presenting ability and T-cell activation and persisting antitumor immunity. In summary, ISL@RLP-Fe is a carrier-free nanodrug composed of active materials (RLP, ISL, Fe^3+^), so there are no inert carriers or associated biosafety concerns. It is achieved through ultrasonic-assisted self-assembly in a mild environment (chemically unmodified, heating-free, and anaerobic-free process) with solely natural ingredients, and the zebrafish embryo assay proves its good biocompatibility. This system combines the synergistic effects of chemotherapy, CDT, and immunotherapy, successfully exerting the “1 + 1 + 1 > 3” antitumor effect. This strategy provides a potential paradigm for multifunctional nanodrugs developed from the resources of traditional Chinese medicine in anticancer therapy. Future studies will be conducted on optimal formulation design, higher ISL loading, and further demonstration of the RLP-mediated immune stimulation mechanism.

The therapeutic efficacy and safety profile of ISL@RLP-Fe require rigorous validation across a broader spectrum of preclinical models as well as in subsequent clinical trials. While promising preclinical data have been reported, translational confidence remains limited without comprehensive cross-model reproducibility and human pharmacokinetic/pharmacodynamic characterization. Critically, the clinical advancement of nanoplatforms hinges on the establishment of a harmonized, internationally aligned evaluation framework comprising standardized protocols for physicochemical characterization (e.g., size distribution, surface charge, colloidal stability, drug loading efficiency, and release kinetics), systematic biocompatibility assessment (including hemocompatibility, complement activation, and endothelial barrier integrity), and tiered toxicological profiling (acute/chronic toxicity, genotoxicity, immunotoxicity, and biodistribution-dependent off-target effects). Equally indispensable are robust, longitudinal monitoring mechanisms integrated into early-phase clinical development: prospective, multi-omics-informed follow-up studies must track immunomodulatory outcomes (e.g., cytokine dynamics, T-cell repertoire diversity, macrophage polarization states), functional biomarkers of major organ systems (e.g., hepatic transaminases, renal eGFR, cardiac troponins), and oncologic endpoints, including minimal residual disease status, time-to-progression, and site-specific recurrence patterns. Such a holistic, mechanism-driven, and regulatory-grade validation strategy is essential not only to de-risk clinical translation but also to inform rational design iterations, enable meaningful benefit–risk assessments, and ultimately support evidence-based regulatory decision-making for next-generation nanotherapeutics.

### 2.2. Mangiferin-Based Carrier-Free Nanomedicine Against Ptx-Resistant Breast Cancer

The TME is intricate and constantly adaptive to its surroundings, providing the primary means by which cancer cells can survive death caused by available conventional approaches after their metastasis, promoting drug resistance [82,83]. Knowledge of the features of the TME and resistance has also encouraged nanomedicine design to remodel the TME to overcome drug resistance [84]. Mangiferin (MGF), a widely used natural C-glycosyl xanthone present in mangoes and honeybush tea, has a history of traditional medicinal use, and recent preclinical studies are now searching to boost the spectrum of therapeutic activities in preclinical studies [85,86]. MGF, a dietary supplement, has a good safety profile at the preclinical level and is tolerated up to 2000 mg/kg [87,88]. MGF induces multiple resistance factors that interfere with cytokine production (TGF-β, IL-6) and block STAT3, leading to the destruction of the protective TME and re-sensitization of resistant cells to chemotherapy [89,90]. MGF inhibits TGF-β and IL-6, two key cytokines produced by cancer-associated fibroblasts (CAFs) and cancer cells [91,92]. These cytokines favor a pro-inflammatory TME that sustains angiogenesis, metastasis, and chemoresistance. MGF downregulates IL-6 to block the IL-6/STAT3 IL-6–STAT3 axis. IL-6-activation of STAT3 phosphorylation (p-STAT3) is an essential mechanism of chemoresistance in ovarian and gastric cancers. MGF impairs the expression of p-STAT3, thus blocking the pro-survival signal not only in cancers, but also in CAFs [93]. MGF selectively induces ferroptosis, an iron-mediated process of cell death, in cancer cells via GPX4, thereby representing a promising therapeutic approach as it circumvents other traditional modes of apoptosis; thereby, it can combat a broad range of drug-resistant cancers [94]. Unfortunately, MGF (BSC IV class) is a poorly soluble and permeable drug with low systemic availability, reducing its potency to modulate anti-inflammatory and anticancer effects or induce ferroptosis in vivo [95,96]. This implies that higher doses are necessary for the desired effect, exposing these preparations to additional formulation problems and toxicity.

Zheng et al. [55] developed novel carrier-free, self-assembled MGF-based NPs (MP NPs) via noncovalent interactions, including π–π stacking, hydrogen bonding, and hydrophobic forces. MGF amphiphiles (MMF) are constructed by click chemistry, and they can be self-assembled into NPs (e.g., MP NPs containing paclitaxel) by hydrophobic and hydrogen bonding. The modification of MGF into an amphiphilic (e.g., MMF) group increases the self-assembly potential and drug delivery efficiency. These approaches not only maintain the bioactivities of MGF but also enhance the pharmacokinetic profile for use as nutraceuticals. MMF self-assembled into supramolecular nanofibers with a particle size of 105 nm. Carrier-free MP (MMF/PTX = 10:1) presented a compact filamentous nanostructure with a particle size of ~68 nm. A small NP size (e.g., 68 nm for MP) may improve tumor-penetrating behavior, while stable self-assembly ensures prolonged drug delivery. MP was diluted in PBS, water, and DMEM (with 10% fetal bovine serum); no macroscopic precipitation was observed after 24 h, indicating good stability under physiological conditions. The fluorescence inside the cells was much brighter after cells were treated with MPs than with free ICG, demonstrating the higher uptake efficiency of the nanomedicine. MP (MMF + PTX) functionalized with indocyanine green (ICG) was used to monitor cellular internalization.

The animal experimental data revealed that MP greatly inhibited the proliferation of PTX-resistant 4T1 tumors in Balb/c mice, as confirmed by the notable decrease in tumor size and body tumor weights in the MP treatment group compared with the control group. No noticeable loss of body weight or damage to organs was noted in the MP NP-treated groups, suggesting that the MP NPs possessed good biocompatibility and low toxicity. MP NPs suppressed TGF-β1 and α-SMA, IL-6/STAT3 signaling, and reversed the pro-tumorigenic chemo-resistant inflammatory TME. MP inhibited GPX4 expression in tumors, validating the induction of ferroptosis. Low expression of Ki67 in tumors implies that cancer cell proliferation is reduced. MP overcame PTX resistance by targeting multiple pathways: (1) ferroptosis induction by GPX4 inhibition, (2) CAF function disruption by suppressing TGF-β1/α-SMA, and (3) inhibition of IL-6/STAT3-mediated inflammation, collectively reversing the chemoresistant TME.

These findings demonstrate MP’s multipronged mechanism of MP in reversing chemoresistance. This combined intervention simultaneously inhibits IL-6/STAT3 signaling, inactivates CAFs, and induces ferroptosis, resulting in the reversal of chemoresistance in PTX-resistant breast cancer models. While the study demonstrates that MP reverses chemoresistance through ferroptosis induction and modulation of the inflammatory TME (e.g., IL-6/STAT3 and TGF-β pathways), the interconnected nature of drug resistance mechanisms raises questions about whether targeting these specific pathways alone is sufficient for long-term resistance reversal, even though the authors mentioned that MP “good biocompatibility” in mice and no long-term biodistribution, degradation, nor excretion pathway for the MMF carrier is described, which is critical for the bench-to-bedside translation.

### 2.3. Flavonol-Driven Photothermal Nanodrugs for Robust Chemo-Photothermal Therapy

The development of multimodal targeting platforms is promising for cancer therapy through not only higher efficacy, but also synergetic properties and reduced side effects [97,98]. PTT is a non-invasive, temperature-controllable, and high-spatial cancer therapy that uses light-induced heat to ablate tumors [99,100]. It needs a temperature of over 50 °C to result in tumor ablation; however, such heat leads to damage of normal tissues in the vicinity [101]. Mild hyperthermia (42–49 °C) can induce thermoresistance by upregulating heat shock proteins and inhibiting apoptosis. Organic photothermal nanodrugs are safer and more degradable than inorganics, but they are hindered by hydrophobicity and carrier toxicity; thus, carrier-free nanodrugs consisting of self- or co-assembly are attractive [102,103]. Flavonols (e.g., kaempferol, quercetin, and myricetin) have been found to induce antioxidant, anticancer, anti-inflammatory, and immune-boosting effects [104,105], and improve PTT by disrupting HSP70 and quenching ROS; however, they have weak solubility and limited bioavailability [106,107]. Co-assembling flavonols with photothermal molecules can increase the efficiency and safety of chemotherapy and photothermal therapy. Perylene diimide photothermal agents offer potent photothermal conversion because J-aggregates permit NIR absorption, but rigid conjugation favors H-aggregates, restricting biomedical applications [108,109]. Flavonols supply hydrogen bond sites to guide perylene diimide assembly.

**Figure 4:**
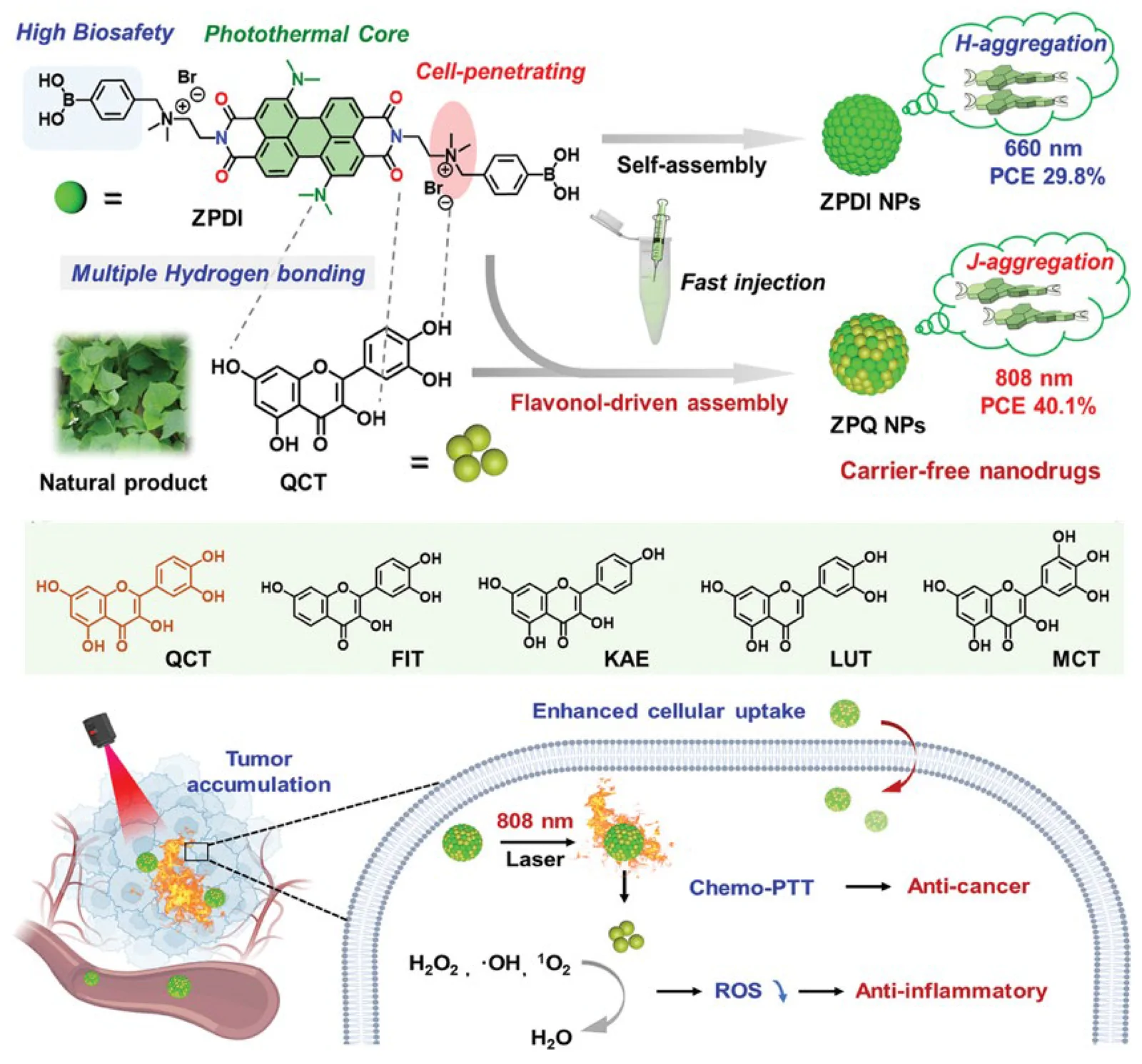
Schematic of flavonol-driven photothermal ZPQ NPs to enhance chemo-photothermal combined therapy. Reproduced from Ref. (Fan et al., 2024) [110] with permission from © 2024 Wiley-VCH GmbH.

Inspired by the abovementioned key points, Fan et al. [110] synthesized an amine-substituted photothermal agent, ZPDI, and constructed carrier-free nanomedicines (ZPQ NPs) via flavonol-mediated co-assembly (Figure 4). ZPQ NPs are carrier-free NPs formed by direct co-assembly of ZPDI and flavonols (e.g., Que), achieving drug loading up to 60% because both components form the nanoparticle framework. Que’s dimethylamine groups engage in H-bonding with ZPDI hydroxyls, π–π stacking stabilizes the core, and ZPDI’s phenylboronic acid groups confer zwitterionic properties for good solubility and biocompatibility. Optimized at a ZPDI:QCT ratio of 1:4, ZPQ NPs were 25 nm (vs 95.6 nm for pure ZPDI), with excellent thermal and physiological stability. J-aggregate formation shifted the absorption to ~ 750 nm, enhancing the polycarboxylate ether (PCE) for effective NIR PTT with minimal side effects. In summary, flavonol-promoted carrier-free assembly enhances loading and biocompatibility owing to specific interactions and tunable nanostructures. At 660 nm, ZPDI NPs and ZPQ NPs heat above 50 °C; ZPDI NPs reach a slightly higher temperature due to stronger 660 nm absorption, but ZPQ NPs exhibit a higher photothermal conversion efficiency (PCE). Under 808 nm, ZPQ NPs show a marked temperature rise (~60 °C in 10 min at 1.0 W cm^−2^) due to J-aggregation-induced red-shifted absorption, with a PCE of 40.1% and superior performance to ZPDI NPs (weaker 808 nm response from H-aggregates). Looser J-aggregation in ZPQ NPs facilitates TICT, resulting in increased heat generation. Hence, ZPQ NPs plus laser attained higher anticancer efficacy (≈67.29% apoptosis and 3.87% necrosis) than ZPDI NPs by inhibiting HSP70.

ZPQ NPs hinder HSP70 expression and weaken the heat-shock resistance of cancer cells, leading to augmentation of cancer cell sensitivity to photothermal therapy. This downregulation raises apoptosis to ~67.3% after treatment, opening the pathway to the effectiveness of chemo-photothermal synergetic therapy. ZPQ NPs efficiently accumulated in 4T1 tumors, with the fluorescence peaking at 1 h and persisting for 10 h. They provide clearer tumor signals and a higher SNR than ZPDI NPs, likely owing to enhanced 4T1 uptake. Tumor-specific accumulation at 2 h via ex vivo imaging (due to their size) and rapid hepatic and renal clearance were demonstrated. In healthy mice, 5 mg/kg caused no significant long-term toxicity over a 7–14-day period, with normal histology, CBCs, and liver/kidney biochemistry. In summary, ZPQ NPs exhibited minimal biotoxicity, high biocompatibility, rapid clearance, and no detectable organ or systemic toxicity. Overall, although current photothermal agents show high conversion efficiency, their clinical translation is hindered by uncertain metabolism and potential toxicity.

However, further in-depth investigations are still warranted regarding reproducibility and clinical translatability. First, systematic studies on the scale-up of manufacturing processes remain lacking. Carrier-free nanomedicines prepared in a laboratory are typically produced in small batches (on the milligram scale); however, scaling up to production-relevant quantities (gram- to kilogram-scale) presents unresolved challenges, including inadequate mixing homogeneity, suboptimal heat transfer efficiency, inefficient solvent recovery, and poor batch-to-batch consistency. Moreover, the absence of protective carrier materials renders the self-assembly behavior of drug molecules particularly susceptible to process perturbations during scale-up, thereby imposing additional technical hurdles. Second, exploration of administration routes remains insufficiently diversified. Current research predominantly focuses on intravenous delivery, while applicability to alternative routes—such as oral or topical administration—has been inadequately investigated. Given that flavonol exhibits extremely low oral bioavailability, it remains unclear whether carrier-free nanomedicines can substantially enhance their intestinal absorption, and how nanoscale architecture maintains structural integrity within the complex gastrointestinal environment following oral administration; both questions necessitate rigorous further investigation.

### 2.4. Egcg-Based Multifunctional Carrier-Free Nanodrug to Enhance Cancer Therapy

Bladder cancer is a common malignancy of the urinary tract [111]. Although immunotherapy can alleviate clinical signs of bladder cancer, only 20–45% of patients respond to immunotherapy; thus, many patients need combination strategies [112,113]. Ferroptosis can sensitize tumors to immunotherapy, but ferroptosis inducers are limited by poor tumor pharmacokinetics and accumulation [114,115]. (-)-Epigallocatechin gallate (EGCG), the water-soluble ester of 2-hydroxybenzopyran and gallic acid, is the principal green tea polyphenol with health benefits, including antioxidant [116], anti-inflammatory [117], antiviral [118], and antitumor effects [119], making it a nutraceutical candidate for functional/health foods. However, EGCG is unstable in solution/air due to the presence of many hydrogen-bond donors and receptors, and can appear light pink, limiting its clinical applications. Its pronounced bitterness also limits its consumer acceptance. Bitterness is detectable at ~90 mg/L; ~300 mg/L is tolerable in foods, while ~1800 mg/L is extremely strong [120,121].

**Figure 5:**
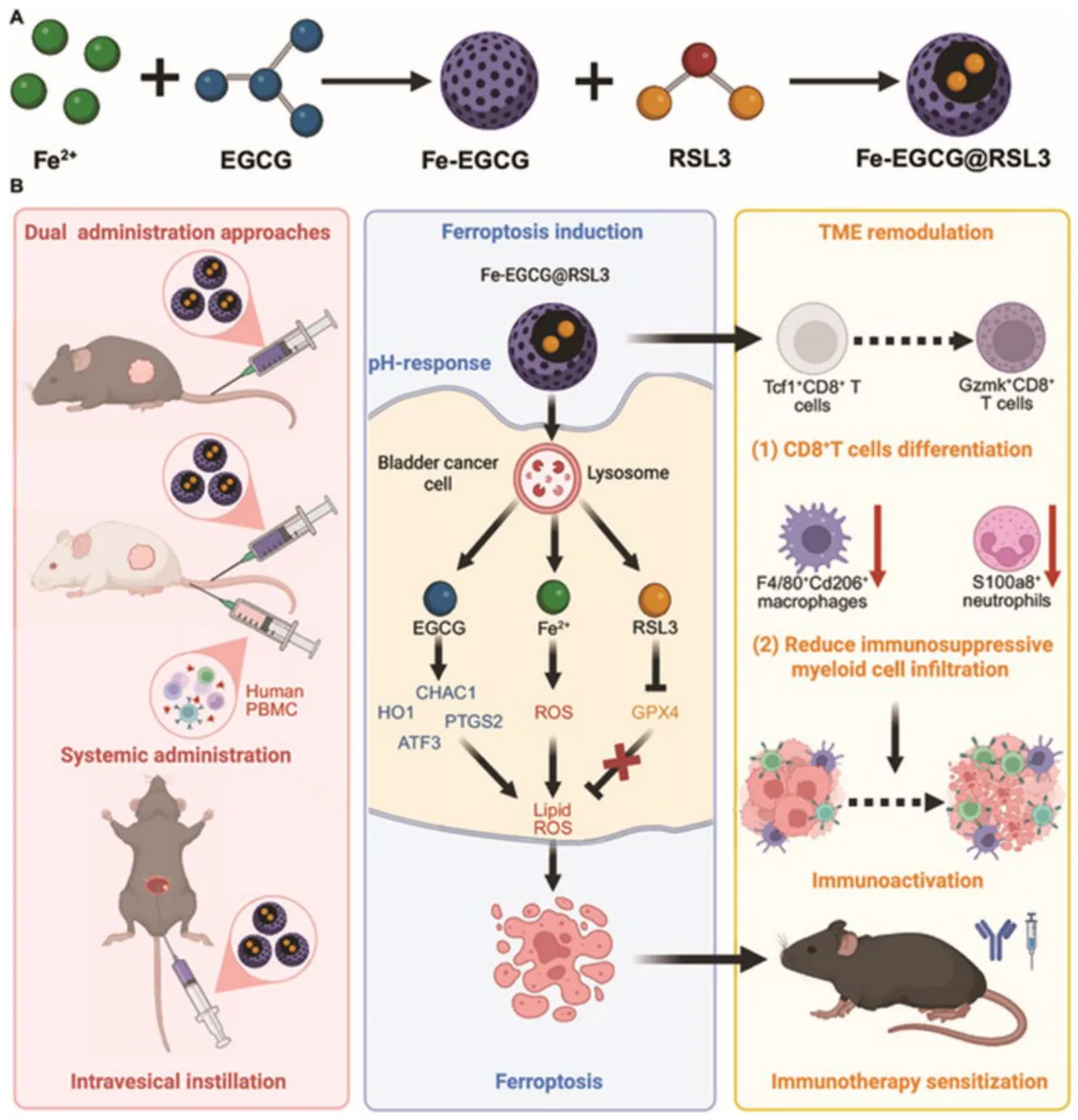
Schematic of multifunctional nanodrug Fe-EGCG@RSL3 for enhanced bladder cancer immunotherapy. (**A**) Schematic illustration of the preparation of the nanovehicle Fe-EGCG and the nanomedicine Fe-EGCG@RSL3; (**B**) The Fe-EGCG@RSL3 nanomedicine induces ferroptosis in bladder cancer via multiple synergistic mechanisms. Reproduced from Ref. (Zhang et al., 2025) [56] with permission from © 2025 Zhang et al.

Zhang et al. [56] designed a multimodal nanomedicine, Fe-EGCG@RSL3, composed of Fe^2+^, EGCG, and the ferroptosis inducer RSL3, which enhanced ferroptosis and improved bladder cancer immunotherapy effects (Figure 5). Initially, among the six antitumor polyphenols, the EGCG generated the most potent ferroptosis-sensitizing effects in both human and mouse bladder cancer cells, while having a negligible impact on normal urothelial cells. Zhang et al. [56] prepared Fe-EGCG@RSL3 by the rapid nano-complexation of EGCG, Fe^2+^, and RSL3 (Figure 5A). Fe-EGCG@RSL3 disintegrates in an acidic environment (e.g., in tumor lysosomes) and can function as a controlled drug delivery system. The pH-responsive design ensures that the EGCG is stabilized in circulation and activated in the TME, while further stabilizing the nanomedicine via Fe^2+^ coordination and hydrophobic interaction with RSL3, which helps prevent the ECGC from losing structural stability at physiological pH. Intravesical instillation eliminates systemic degradation and enhances stability and tumor-specific accumulation. The complex entered bladder cancer cells and released Fe^2+^ in a pH-dependent manner, indicating pH-responsive drug delivery (Figure 5B).

Fe-EGCG@RSL3, at an equivalent low-dose RSL3, significantly enhanced the levels of lipid peroxides and ROS to induce ferroptosis in bladder cancer cells and exhibited low toxicity to normal urothelial cells. The physical Fe^2+^/EGCG mixture without nano-assembly was far less efficient, demonstrating that the nanocarrier structure is critical for synergism. Systematic tail-vein delivery of Fe-EGCG@RSL3 in immunocompetent mice or human PBMC-reconstituted immuno-deficient mice metastasizing to bladder tumors results in monotherapy efficacy matching that of anti-PD-1. The combination of Fe-EGCG@RSL3 and anti-PD-1 significantly inhibited tumor growth, demonstrating enhanced antitumor effect. 4-HNE IHC showed enhanced ferroptosis in tumors treated with a non-systemic toxicity phenomenon.

Fe-EGCG@RSL3 was also exploited for accessibility to the bladder for local therapy. Fe-EGCG@RSL3 via bladder perfusion as described by Zhang et al. [56] was tested on an orthotopic bladder cancer model in immunocompetent mice. The Fe-EGCG@RSL3 monotherapy delayed tumor growth, and Fe-EGCG@RSL3 conjugation with anti-PD-1 mAb showed a striking growth-inhibitory effect that prolonged survival, indicating enhanced antitumor effect (Figure 5B). This finding indicates that Fe-EGCG@RSL3 can be effectively administered via multiple routes to enhance immunotherapy. Single-cell examination of mouse tumors indicated that the naive CD8^+^ T (Lef1^+^) decreased, whereas the effector memory CD8^+^ T (Gzmk^+^) increased in the nanodrug plus anti-PD-1 group, indicating that Fe-EGCG@RSL3 provokes naive-to-effector memory differentiation. Collectively, Fe-EGCG@RSL3 may boost antitumor CD8^+^ T-cell function by promoting differentiation towards effector and effector memory states. Further single-cell analysis of myeloid subpopulations showed a significant reduction in M2-like Folr2^+^ macrophages and neutrophil subsets in the combination group, which indicated that Fe-EGCG@RSL3 improved antitumor immunity by decreasing the infiltration of immunosuppressive macrophages and neutrophils. The Fe-EGCG@RSL3 nanomedicine accumulated and induced ferroptosis synergistically with RSL3 in tumor tissues. It provides a highly targeted, low-toxicity delivery, systematic change in TME, and various administration approaches to bring a new type of ferroptosis-immunotherapy approach for clinical bladder cancer treatment and potentially enhances immunotherapy response.

Intelligence is conferred through rationally engineered stimulus-responsiveness: the nanoplatform exhibits precise spatiotemporal control over drug release and functional activation in response to endogenous or externally applied triggers. For instance, pH-labile glycosidic linkages or ortho-quinone moieties enable selective disassembly and payload liberation within acidic tumor microenvironments or lysosomal compartments; ROS-sensitive boronic ester modifications facilitate on-demand antioxidant amplification in inflamed or ischemic tissues; and near-infrared (NIR) light-responsive flavonoid–metal coordination complexes permit noninvasive, spatially confined photothermal or photodynamic therapy. Furthermore, surface functionalization with targeting ligands (e.g., folic acid, RGD peptides, or aptamers) can be achieved without compromising structural integrity, enabling active tumor homing and receptor-mediated transcytosis across biological barriers such as the blood–brain barrier.

## 3. (Poly)phenol-Based Carrier-Free Nanoplatform

(Poly)phenols constitute a large and structurally diverse class of plant-derived secondary metabolites characterized by the presence of (multiple) phenolic hydroxyl groups attached to one or more aromatic benzene rings. They are ubiquitously distributed across the plant kingdom, and particularly abundant in edible botanical sources such as fruits, vegetables, legumes, cereals, tea leaves, coffee beans, cocoa, and seeds, as well as in traditional medicinal herbs used globally in phytotherapy and ethnopharmacology. Critically, the inherent amphiphilic architecture of polyphenols, featuring polar, hydrogen-bond-donating phenolic hydroxyl groups and nonpolar, π-electron-rich aromatic rings, confers unique molecular functionality beyond mere payload encapsulation. This dual character enables polyphenols to serve not only as active pharmaceutical ingredients but also as integral structural and functional components within delivery platforms: their hydrophilic moieties engage in hydrogen bonding, electrostatic interactions, or covalent conjugation with drug molecules or targeting ligands; meanwhile, their hydrophobic aromatic domains drive spontaneous self-assembly, stabilize nanoparticle cores via π–π stacking and hydrophobic interactions, and mediate affinity for lipid bilayers or hydrophobic binding pockets in proteins and membranes. Such “dual role” behavior significantly enhances formulation robustness, loading efficiency, colloidal stability, and biological performance.

### 3.1. Erianin- and Curcumin-Based “Triadic” Platform for Drug Delivery

*Dendrobium candidum* is a well-known plant that has been used as a nutritional food ingredient or functional beverage for thousands of years in China [122,123]. It can nourish the stomach, balance intestinal flora, boost immune function, clear fever, and reduce blood pressure [124,125]. Erianin (ERI), a bibenzyl extracted from *Dendrobium candidum*, is an ideal drug candidate with therapeutic potential [126,127]. Its broad pharmaceutical use and other desirable bioactivities such as antitumor, antiviral, anti-inflammatory, and antioxidation properties make it a promising lead compound for drug development [128,129]. However, ERI has poor solubility, fast metabolism, and low bioavailability, which limits its clinical translation [130,131]. Carrier-free nanomedicine holds great promise, as it offers a potentially efficacious approach to improve dissolution and systemic exposure, further increasing the therapeutic efficiency, and does not carry the related concerns of carriers in safety [132]. Cur, a bioactive polyphenol derived from *Curcuma longa* (turmeric), is a bright yellow pigment with broad pharmacological activities, including antioxidant [133], anti-inflammatory [134], anticarcinogenic [135], and antiangiogenic [136] effects. These effects arise from the modulation of multiple molecular targets and signaling pathways (e.g., NF-κB, Nrf2, STAT3, COX-2, and MAPKs), and through the regulation of apoptosis, cell cycle, and angiogenesis [137,138]. Nevertheless, Cur faces major clinical translation challenges due to its poor aqueous solubility, rapid metabolism, and chemical instability under physiological conditions, which leads to low systemic bioavailability [139,140]. Thus, different formulation strategies have been investigated for targeted improvement of solubility, stability, and delivery, such as liposomes [141], polymeric NPs [142], micelles [143], prodrugs [144], conjugates [145], and co-delivery with bioenhancers.

**Figure 6:**
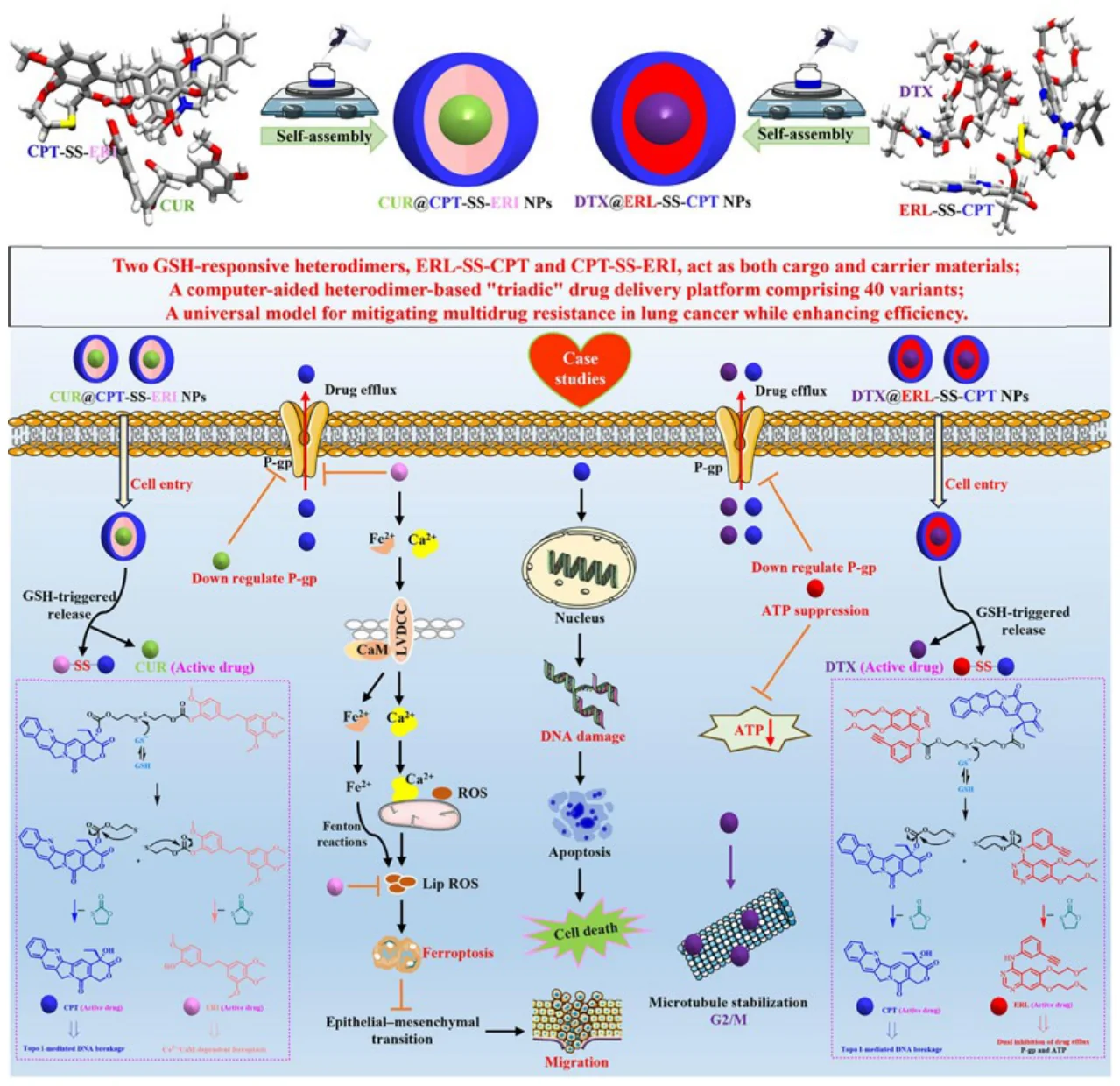
Schematic of carrier-free drug delivery platform CUR@CPT-SS-ERI NPs to enhance anticancer efficiency. Reproduced from Ref. (Yang et al., 2025) [57] with permission from © 2025 Elsevier B.V.

Lung cancer is the leading cause of cancer mortality worldwide and remains the deadliest cancer globally [146,147]. Multidrug resistance (MDR), a critical and widespread problem in the context of lung cancer chemotherapy, persists as the leading obstacle in the effective treatment of lung cancer [148,149]. It is widely recognized that multitarget phytochemicals can sensitize lung cancer cells to conventional chemotherapy [150]. As mentioned above, to reduce MDR in lung cancer cells, we developed a computer-aided “triadic” drug delivery platform that was targeted to improve therapeutic efficacy (Figure 6) [57]. Initially, we designed and synthesized the GSH-responsive heterodimer CPT-SS-ERI as carrier and payload materials, then established a “triadic” platform with 40 variants based on the heterodimer, and did a case study with CPTSS-ERI loaded with CUR (CUR@CPTSS-ERI NPs). The average hydrodynamic diameter of CPTSS-ERI NPs was 94 nm. After CUR encapsulation, particle size increased to 108 nm. After incubation with 10 mM GSH, the particle size of CUR@CPT-SS-ERI NPs increased to 1538 nm, showing the cleavage of disulfide bonds and controlled drug release. Size expansion under the influence of glutathione in the TME promotes drug release, and normal physiological conditions maintain stability (for example, the size change is negligible at 10 μM GSH). In summary, these strategies and results emphasize the contribution of optimized drug load capacity and stability, along with their stimulus-responsive release, in improving the therapeutic potential against MDR in lung cancer. Multiple noncovalent forces (π–π stacking, hydrogen bonding, hydrophobic interactions, and sulfur bonds) underlie this platform for self-assembly. After internalization by cells, such NPs are exposed to high levels of GSH for disulfide bond cleavage, increasing the release of drugs, increasing the water solubility of the drug, lowering the dosage of free drugs, and intracellular accumulation. The MTT assay results showed that the CUR@CPT-SS-ERI NPs had good biocompatibility in normal BEAS-2B cells. At all tested concentrations (0.0625 to 20 μM), the viability of BEAS-2B cells remained above 80%, indicating minimal cytotoxicity to normal cells. This demonstrates that the normalized cell toxicity of CUR@CPT-SS-ERI NPs was nearly the same as that of CPT-SS-ERI NPs, whereas CPT caused substantial dose-dependent toxicity. This result implies that CUR@CPT-SS-ERI NPs are safe because they avoid adverse toxic effects on normal cells by a larger margin than free drugs. The ability of NPs to maintain high viability in healthy cells while showing increased cytotoxicity in tumor cells due to GSH-responsive drug release demonstrates their selective therapeutics. The selective cytotoxicity and lowered off-target effects indicates that NPs are safe for clinical trials. Moreover, they inhibit P-gp, eventually leading to cell death.

In summary, we developed a three-in-one self-delivery system integrating three parts (e.g., CUR) into CPT-SS-ERI connected by a disulfide bond. CPT-SS-ERI served as the drug cargo and carrier itself (to minimize external carriers) and provided a high content of drug loading rate (76.1% *w*/*w*). The cleavage of the disulfide bond in the TME (high GSH concentration) facilitated controllable drug release. The high cellular uptake rate, cell growth inhibition, inhibition of P-gp expression, and sensitization of Taxol-resistant cells using NPs overcame MDR. The platform contained 40 variants and was flexible for drug combinations (i.e., CUR@CPT-SSERI NPs). In vitro tests (i.e., cytotoxicity in A549 and BEAS-2B cells) can be considered efficient; however, the in vivo efficacy and long-term safety in vivo efficacy need to be validated. The used lung cancer A549 and normal bronchial BEAS-2B cells were tested in vitro; their range of applications to various kinds of cancers and tumors with resistance is to be demonstrated. The physiological stability for a prolonged period and its biodegradability has not been reported; it is unclear whether storage stability is indicated.

Future: One strategic approach involves the rational design and development of next-generation optical agents endowed with enhanced antitumor efficacy, specifically engineered to concurrently alleviate tumor hypoxia, selectively disrupt aberrant tumor vasculature, and induce controlled, localized hyperthermia within the tumor microenvironment. These multifunctional agents operate through photophysical and photochemical mechanisms upon precise spatiotemporal irradiation with near-infrared (NIR) or visible light. By normalizing oxygen tension, such agents mitigate hypoxia-driven immunosuppression, including HIF-1α-mediated upregulation of PD-L1, adenosine accumulation, and myeloid-derived suppressor cell infiltration, thereby restoring dendritic cell maturation and T-cell effector function. Meanwhile, rigorous translational validation, including pharmacokinetic/pharmacodynamic modeling, immune monitoring via multiparametric flow cytometry and single-cell RNA sequencing, and longitudinal assessment of T-cell receptor clonality, is essential to optimize dosing regimens, irradiation parameters, and sequencing strategies to maximize therapeutic index while minimizing off-target immune toxicity.

### 3.2. Rosmarinic Acid-Based Carrier-Free Network to Enhance Cancer Nanotherapy

*Perilla frutescens* (L.) is an annual Lamiaceae herb widely grown in China, Korea, India, and Nepal [151]. It has a distinct aroma and is used as a spice, vegetable, fragrance, and natural colorant [152]. It is used medicinally for its cardiovascular health [153], anti-inflammatory [154], anti-rheumatic [155], and antidepressant benefits [156]. Dried leaves, stems, and seeds of *P. frutescens* are mentioned in the 2025 *Chinese Pharmacopeia* as a traditional Chinese medicine (TCM) for asthma, cough, influenza, and vomiting. Rosmarinic acid (RA), a phenolic acid isolated from *P. frutescens*, has antitumor effects, but due to the presence of a tumor acidic microenvironment and high levels of GSH, tumor resistance to RA monotherapy weakens its effectiveness [157]. Inspiringly, many different physiological and biochemical processes depend on essential trace metals; metal–polyphenol networks assembled by metal ions have multifunctional biofunctions and are widely studied for disease diagnosis and treatments [158].

**Figure 7:**
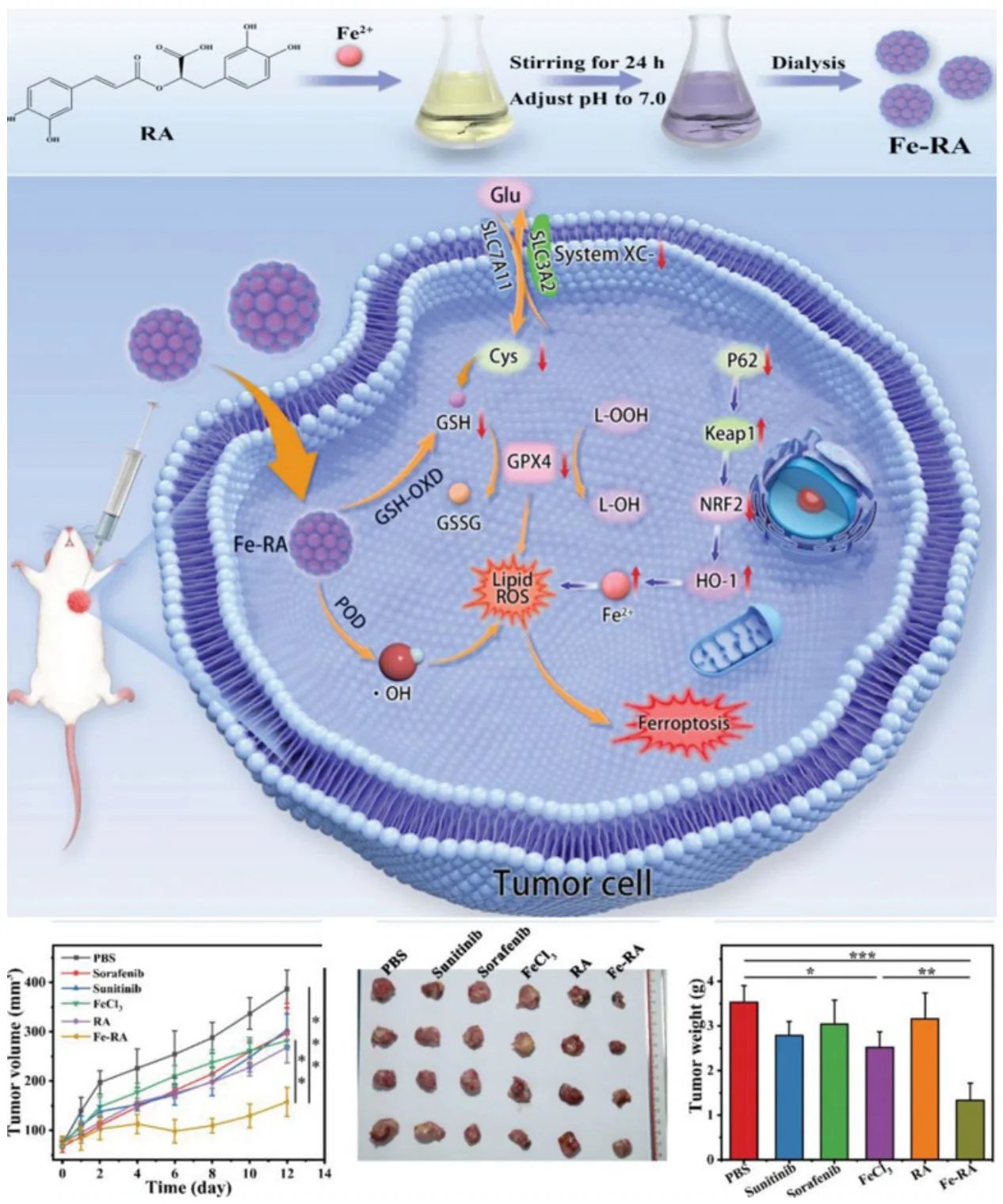
Schematic of carrier-free drug delivery platform Fe-RA to enhance ferroptosis-based cancer therapy. * represents *p* < 0.05, ** represents *p* < 0.01, *** represents *p* < 0.001. Reproduced from Ref. (Chen et al., 2024) [58] with permission from © 2024 Wiley-VCH GmbH.

Chen et al. [58] designed a self-assembled nanocomposite (Fe–RA) using vicinal dihydroxy and carboxyl groups to overcome the monotherapy limitations (Figure 7). During Fe-RA synthesis, RA acts as both a reducing and chelating agent, reducing Fe^3+^ and enabling Fe–O coordination with RA. The adjacent phenolic hydroxyls on RA chelate Fe^3+^ via Fe–O bonds, forming a tetrahedral Fe–O4 coordination framework and self-assembled network. The Fe-RA nanoparticle size is determined by Fe^3+^–RA coordination, resulting in Fe–O bonds. As measured by DLS, the hydrated diameter is ~316 nm, which is larger than the ~200 nm dry SEM size because of the hydration layer of the solvents. The size is controlled by the coordination chemistry and synthesis parameters (Fe^3+^/RA ratio and solvent), providing excellent colloidal stability characterized by a high zeta potential (~ –45 mV). In acidic TME, Fe^2+^/Fe^3+^ cycling produces •OH via Fenton reactions, and peroxidase-like action of RA converts H_2_O_2_ to •OH, increasing ROS and ferroptosis. Fe^3+^ depletes intracellular GSH and, in combination with GPX4 inhibition, weakens antioxidant defense to increase ferroptosis. Depletion of GSH also preserves RA bioavailability. RA chelation stabilizes Fe^2+^, increases cellular iron, and disrupts iron metabolism to induce ferroptosis. Furthermore, RA also reduces tumor growth by blocking NF-κB signaling and chemokine receptors (e.g., CCL11/CCR3) through β-kinase-related pathways, which complement ferroptosis, allowing RA to enter tumors more efficiently to gain Fe-RA with Fe^2+^ and RA at the same time. Overall, ROS overproduction, GSH depletion, and GPX4 inactivation form a “dual strike” on tumor cells and facilitate resistance bypassing for apoptosis-based therapeutics.

Mechanistically, Fe-RA downregulates GPX4 and System Xc-, disturbs the NRF2/P62/Keap1 axis, and attenuates intracellular GSH synthesis to boost tumor cell sensitivity to iron and drive ferroptosis. Utilizing synthetic iron–polyphenol network catalysis and the availability of TME cues, it synergistically induces ferroptosis for efficient tumor treatment. Second, high-valence TMI (Fe^3+^, Cu^2+^) is reduced by GSH, leading to GSH depletion, and Fe^2+^ and Cu^+^ can enhance the Fenton reaction to produce extra ROS in cells to upregulate intracellular ROS to achieve ferroptosis or cuproptosis. The depletion of GSH through this mechanism is contingent on the supply of metal ions and redox equilibria. Combining the flexible metal-ion delivery network with other GSH-uptaking agents–bimetal/trimetal phenolic networks–may achieve a combined GSH-depletion strategy that can effectively trigger triple or quadruple antitumor mechanisms, which makes the associated study deserving of exploration.

Future research endeavors must therefore adopt a multidisciplinary, iterative, and translationally oriented approach. First, assembly conditions, including solvent composition, temperature ramping profiles, mixing kinetics, purification methodologies, and lyophilization stabilizers, require systematic optimization to achieve narrow polydispersity indices (<0.15), high encapsulation efficiency (>90%), and long-term colloidal stability (>6 months at 4 °C). Second, component stoichiometry, particularly the molar ratio of photosensitizer/photothermal agent to chemotherapeutic prodrug or conjugate, must be fine-tuned to balance dual functionality: sufficient photothermal conversion efficiency (PCE ≥ 40% under clinically relevant 808 nm irradiation) while preserving cytotoxic potency and minimizing dark toxicity. Third, rigorous efficacy and safety assessment must extend beyond conventional subcutaneous xenograft models to encompass orthotopic, metastatic, patient-derived xenograft, and genetically engineered mouse models that recapitulate human tumor pathophysiology, stromal interactions, and immune contexture. Longitudinal studies should integrate multimodal imaging to quantitatively track spatiotemporal biodistribution, real-time thermal mapping during laser irradiation, and dynamic drug release profiles. Comprehensive toxicology evaluations should also be done, including repeated-dose systemic toxicity, immunotoxicity, genotoxicity, and organ-specific histopathology.

### 3.3. Capsaicin-Based “Three Birds with One Stone” Carrier-Free Drug Delivery System

Breast cancer metastasis is difficult to treat, and targeted inhibitors (angiogenesis, MMPs, and VEGFRs) provide limited benefits [159]. Given that CTCs, but especially CTC clusters, drive metastasis—with clusters up to ~50-fold metastatic potential compared with individual CTCs—inhibiting CTC formation may be an effective approach, and that combination therapy, delivering cytotoxic therapy along with CTC inhibitors, may result in the control of both primary tumor growth and metastatic dissemination [160,161]. Gemcitabine (Gem) is a first-line anticancer drug that inhibits DNA synthesis and causes apoptosis; however, its response rate is modest, highlighting the need for better efficacy [162,163]. Therefore, in many cases, it has been used in combination with radiotherapy [164], PDT [165], and PTT [166]. Chemo–PDT combination therapies were most efficacious, since the ROS produced in PDT break into organelles and damage DNA, thus damaging repair and causing the loss of chemoresistance. Capsaicin (Cap) is a representative capsaicinoid compound in chili pepper that can activate TRPV1 and has analgesic [167], anticancer [168], antioxidant [169], anti-inflammatory [170], and other metabolic effects. It can be used in weight loss products because it can increase energy consumption and reduce body fat percentage [171,172]. Cap can be incorporated to inhibit CTC and metastasis through TRPV1-mediated calcium signaling [173]. Cyclometalated iridium(III) complexes are promising phototherapeutic agents [174]. However, free drugs exhibit systemic toxicity and poor pharmacokinetics, but nanomedicine offers a solution.

With the above considerations in mind, Shan et al. developed a self-assembling carrier-free system (Ir-Gem@Cap) [59]. Hydrophilic gemcitabine and a hydrophobic Ir(III) photosensitizer were linked by an alkyl ester to form the prodrug M-Ir-Gem, enabling the co-delivery of capsaicin. An optimal cap-to-M-Ir-Gem ratio of 10 mg Cap to 12.9 mg M-Ir-Gem was identified, yielding a DLC of 20.0% and EE of 32.3%. This composition ensures stable NPs (PDI < 0.3) and pH-responsive drug release, enhances targeted delivery, and reduces side effects. Without TP irradiation, Ir-Gem has an IC_50_ of 18.47 μM, reducing to 3.31 μM with TP. Ir-Gem@Cap showed higher cytotoxicity than Ir-Gem without TP (IC_50_ 7.31 μM) and was further improved with TP (IC_50_ 2.51 μM). Cell viability declined with increasing concentration and was greatest for Ir-Gem@Cap + TP. Ir-Gem@Cap was more potent at lower doses than Ir-Gem because of the combined effects of gemcitabine, the Ir(III) complex, and capsaicin. TP irradiation enhanced the efficacy of both, yielding ~5.6-fold and ~2.9-fold IC_50_ reductions for Ir-Gem and Ir-Gem@Cap, respectively. Overall, Ir-Gem@Cap, especially with TP irradiation, enabled potent tumor inhibition at lower doses, with potentially reduced systemic toxicity. Ir-Gem@Cap with TP laser irradiation (800 nm, 50 mW cm^−2^, 120 s) produced the strongest tumor suppression, which was significantly better than that of the non-irradiated group (*p* < 0.05). No significant body weight loss or systemic toxicity were observed, hemolysis amounted to <5%, and liver/kidney function remained normal. In vivo imaging showed tumor accumulation, with luminescence peaking at 6 h and remaining high 24 h post-injection. Ir-Gem@Cap arrested breast cancer proliferation and metastasis via tumor pH-sensitive release, PDT, anti-metastatic effects, and synergistic chemotherapy. Ir-Gem@Cap was stable at physiological pH (7.4) but deaggregated in an acidic TME (pH 5.5) by ester hydrolysis to achieve tumor-targeted drug release. Ir-Gem@Cap exhibits an excellent ^1^O_2_ quantum yield (~72–73%) and two-photon absorption at 800 nm (94.7 GM) for deep-tissue PDT.

It significantly inhibited 4T1 cell migration; two-photon irradiation, 72.2% less migration than control (Gem gives chemotherapy), and endowed anti-metastatic activities by blocking both migration and CTC formation; iridium complex imparted enhanced PDT and tracking effect; the carrier-free structure helped to increase stability and lower off-target toxicity, facilitating efficient targeting of breast cancer cells. Cap encapsulation in Ir-Gem@Cap showed an EE of 32.32% and a DLC of 20.03%, indicating suboptimal loading that may limit efficacy or require higher doses. Metastasis suppression by CTC-targeting agents has been reported in vitro (uptake and cytotoxicity in 4T1 cells) without in vivo metastasis, consequently weakening translational relevance.

It must be noted that current safety assessments cover only 2–4 weeks of dosing, revealing only acute toxicity and failing to detect subchronic or chronic toxicity. Long-term accumulation of the drug or its degradation products, progressive target-organ damage, and delayed toxicity all require observation periods far exceeding the current timeframe. Conventional nonclinical safety assessments typically mandate at least 90-day subchronic and longer-term chronic toxicity studies, far beyond current practice. Moreover, body weight change lacks sensitivity; serum liver/kidney function markers exhibit a “window period” problem—abnormal elevation occurs only after organ injury reaches the threshold and is easily confounded by inter-individual variability; and routine H&E staining detects only overt morphological changes, lacking resolution for early, subcellular damage.

### 3.4. Capsaicin/Curcumin-Based “Three Birds with One Stone” Carrier-Free Drug Delivery System

From the perspective of category classification, the definition criteria for carrier-free-based nanomedicines do not lie in whether the drug molecules have undergone chemical modification, but rather in whether there is a carrier material independent of the active pharmaceutical ingredient within the system. If the building blocks are the drug molecules themselves (regardless of whether they have undergone chemical derivatization), and no additional polymers, lipids, or inorganic materials are introduced as structural supports, it falls within this category. This provides a basis for the application of chemically modified natural products in non-carrier-based nanomedicines and delineates a clear boundary for the design of new delivery systems. Modifying natural products by chemical derivatization can increase their activity, decrease toxicity, and improve bioavailability [175,176]. Natural product-based drug delivery systems provide modifiable sites that can help overcome these limitations. Sulfation [177], acetylation [178], phosphorylation [179], and carboxymethylation [180], the common strategies, can modulate physicochemical properties and biological activities (e.g., antitumor and antiviral), especially the third method, which is simple, inexpensive, biocompatible, and extra plus of being facile. Liu et al. [60] designed a potential carrier-free self-assembled Cap-p@Cur using noncovalent binding. Cap was modified by single-step phosphorylation to yield amphiphilic Cap-p that self-assembles into core–shell nanovesicles in water, with hydrophilic phosphate enhancing dispersibility. The hydrophobic photosensitizer Cur co-self-assembled into Cap-p vesicles to solubilize and stabilize Cur. Cap-p phosphorylation increased amphiphilicity and promoted self-assembly into uniform spherical nanovesicles (~100 nm by SEM), which provided passive tumor targeting. The loading of Cur expanded the size to ~460 nm (DLS) due to hydrophobic forces with Cap-p, while Cap-p alone was ~190 nm (DLS); the expansion of size and PDI ≈ 0.45 is an indicator of modified colloidal stability and release behavior. Both Cap-p and Cap-p@Cur formed well-dispersed nanovesicles in water. Cap-p@Cur remained at <500 nm in H_2_O, DMEM, and PBS after 2 h of storage. The zeta potential remained negative (−10 to −30 mV), which revealed that the formed particles have good stability. Cap-p@Cur presented pH-dependent stability: good stability at physiological pH 7.4, but showed susceptibility to acidic pH 5.0 (tumor-like), with approximately 30% cumulative Cur release at 24 h at pH 5.0, compared to slower Cur release at pH 7.4.

Cap-p@Cur was much more cytotoxic in MDA-MB-231 cells than free Cur or Cap-p, even upon illumination at 3 mW/cm^2^, at 40 μg/mL, up to complete cell death. Light added to the antitumor effect by ROS generation (singlet oxygen and superoxide) was validated using DCFH-DA and DHE. Cap-p remained without any toxicity up to 40 μg/mL, whereas Cap-p@Cur demonstrated an excellent hemocompatibility, with hemolysis below 1.34% at ≤100 μg/mL. Cap-p also showed bone targeting, which may be useful in the treatment of bone metastatic TNBC. Cap-p@Cur encompassed chemotherapy (Cap-p hydrolysis products and Cur) and PDT (Cur’s light-induced ROS). Light irradiation augmented Cur cytotoxicity, leading to tumor-specific killing with fewer off-target effects. Cap-p exhibited good colloidal stability and bone targeting through phosphate groups, but Cap-p@Cur showed time-dependent growth. In addition, Cap-p@Cur also had a broader size distribution (PDI = 0.450) compared to Cap-p (PDI = 0.215), indicating less uniform self-assembly after encapsulating Cur, with a potential effect on reproducibility and therapeutic consistency. Despite adsorbing onto hydroxyapatite, the adsorption only became ~66.1% and ~47.9% at pH 5.0 and pH 6.6, respectively, suggesting low-targeting efficiency towards bone for bone-metastatic TNBC. Free Cur precipitated quickly in water (within 0.5 h); Cap-p@Cur was less stable but only slightly better than Cap-p in terms of colloidal stability, indicating difficulties in stabilizing the hydrophobic drug dispersion. This study was predominantly done in vitro (MDA-MB-231 viability) without evaluation of in vivo pharmacokinetics, biodistribution, or efficacy data, and must be further optimized for stability, uniformity, targeting, and in vivo validation for clinical translation.

To translate this innovative methodology into clinical practice, a comprehensive suite of translational barriers must be rigorously addressed. Chief among these are the reproducibility and robustness of the self-assembly process—critical for ensuring batch-to-batch consistency, scalability, and regulatory compliance. In vivo stability represents another pivotal challenge: nanoscale assemblies must resist premature disassembly or degradation in complex physiological environments characterized by variable pH, enzymatic activity, ionic strength, and protein corona formation. Concurrently, precise control over biodistribution is essential—not only to maximize tumor accumulation, but also to minimize off-target sequestration in reticuloendothelial system (RES) organs such as the liver and spleen. Pharmacokinetic profiling—including absorption, distribution, metabolism, and excretion (ADME) parameters—must be thoroughly characterized across preclinical species to inform first-in-human dosing regimens and predict human clearance pathways. Furthermore, intricate physicochemical and biological interactions between the chemotherapeutic payload and photothermal agent must be systematically elucidated under physiologically relevant conditions (e.g., serum-containing media, tumor interstitial fluid mimics, hypoxic microenvironments). These interactions may modulate drug release kinetics, alter photothermal conversion efficiency, induce unintended synergistic or antagonistic effects, or trigger immunogenic responses. Compounding this complexity is tumor-type-specific heterogeneity; variations in vascular architecture, stromal composition, extracellular matrix density, metabolic profile, and immune cell infiltration significantly influence nanoparticle penetration, retention, and therapeutic response, necessitating stratified evaluation across diverse histological and molecular subtypes.

## 4. Conclusions and Future Perspectives

This review delivers a rigorous, mechanism-informed, and structure–function–activity–outcome-oriented evaluation of polyphenol-based carrier-free smart nanoplatforms. It systematically elucidates how their rationally tunable supramolecular architecture directly dictates colloidal stability, enzymatic resistance, plasma half-life extension, and biodistribution profiles. Critically, we demonstrate that enhanced pharmacokinetic behavior synergizes with intrinsic biological activity: prolonged systemic circulation enables greater accumulation, while surface-functionalized variants achieve active tumor targeting with improved cellular internalization and subcellular trafficking fidelity. Moreover, stimuli-responsiveness triggered by tumor microenvironmental cues such as acidic pH, elevated ROS, or overexpressed enzymes, confers spatiotemporal control over polyphenol release and activation, thereby amplifying therapeutic index and minimizing off-target effects.

**Figure 8:**
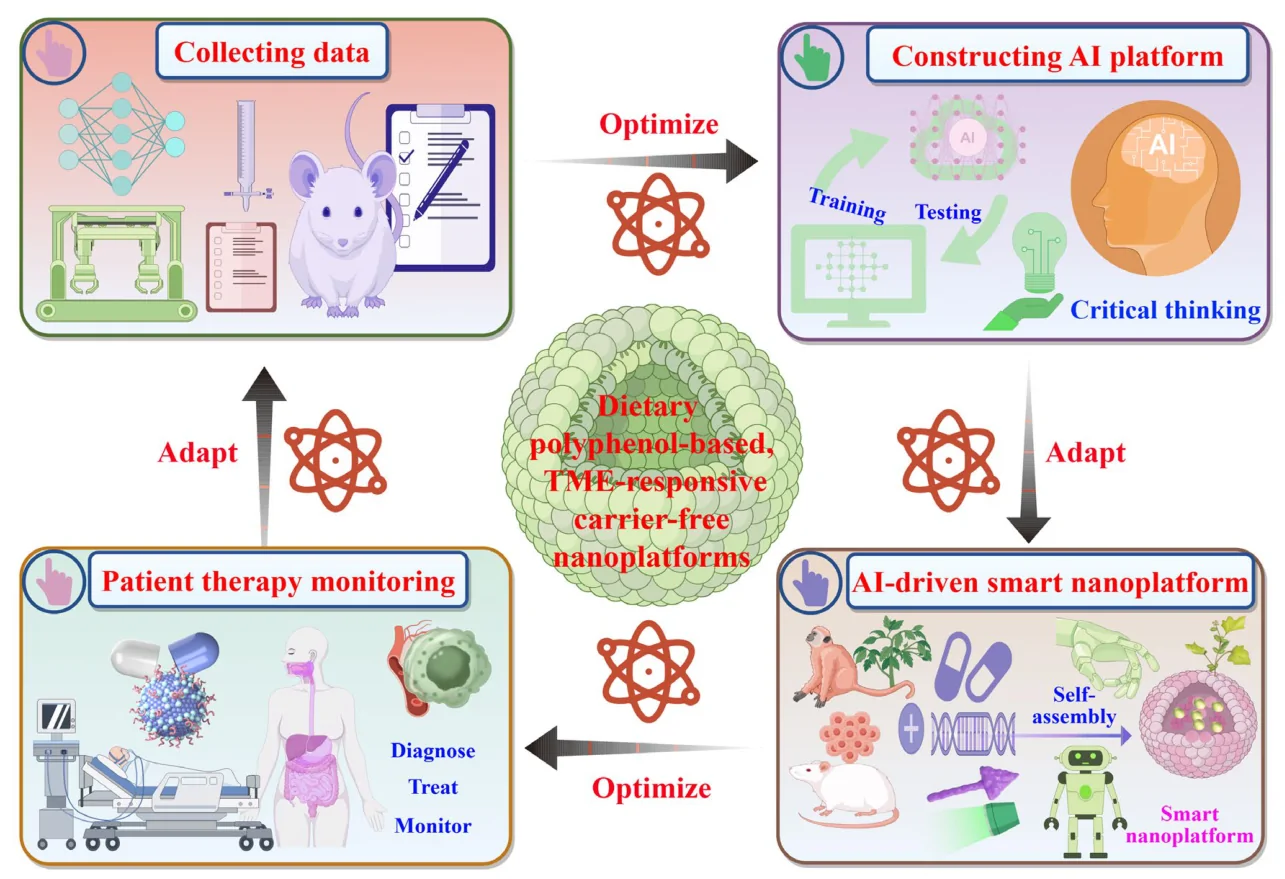
AI-powered polyphenol-based carrier-free nanoplatforms for enhanced clinical translation.

Key scientific hurdles include incomplete understanding of in vivo disassembly kinetics, complex polyphenol–biomembrane interaction mechanisms, and inter-individual variability in polyphenol metabolism mediated by gut microbiota and polymorphic phase II enzymes. From a manufacturing perspective, scalability remains constrained by batch-to-batch reproducibility issues arising from raw material heterogeneity, sensitivity to processing parameters, and lack of robust real-time quality-by-design monitoring tools for nanoscale structural integrity. Regulatory challenges encompass the absence of standardized characterization protocols for carrier-free systems, particularly regarding colloidal stability assessment under physiological stressors (e.g., serum protein adsorption, ionic strength shifts), long-term storage stability validation, and harmonized criteria for defining “nanoscale” in the context of dynamic, non-covalent assemblies. The combination of carrier-free nanomedicines and artificial intelligence, by precisely manufacturing multifunctional nanoparticles, enables simultaneous diagnosis and treatment of diseases, and is fundamentally transforming cancer treatment. Artificial intelligence is being applied to the development of nanoparticles through machine learning, supporting synthesis optimization, modeling and reverse design, accelerating clinical applications and enabling personalized cancer treatment (Figure 8). Furthermore, current preclinical efficacy and safety assessment frameworks inadequately capture the unique pharmacodynamics of self-assembled polyphenols. We propose two high-priority, interdisciplinary directions for future research. Furthermore, we argue that harmonized safety frameworks, along with standardized safety endpoints, are necessary for responsible translation.

**Tackling nanotoxicology issues.** Safety and efficacy are the two major parameters addressed for drug development. The clinical translation of nanomedicine has been severely hampered by safety concerns in clinical trials. Functional dietary-component-based carrier-free nanomedicines tend to have excellent safety profiles, but toxicity profiling is still inconclusive and biased on a limited number of organs. To enable safe clinical translation, studies should extend to the skin, reproductive organs, auditory system, brain, eyes, vasculature, and other metabolically active tissues, particularly with prolonged or repeated dosing. Immune and inflammatory responses should be systematically extended to nanoplatforms and degradation products under realistic clinical exposure. The mouse data alone is insufficient because of interspecies differences; a hierarchical translational plan should comprise larger-animal models and humanized systems, ex vivo human tissues, organoids, and organ-on-a-chip platforms to enhance predictivity. Predictive in silico tools and PBPK models for ADMET, accounting for protein corona and aggregation, should be integrated with high-throughput in vitro and multi-omics readouts.

**A prudent analysis of the enhanced permeability and retention (EPR) effect.** The physicochemical properties of nanomedicines profoundly influence the efficacy of the EPR effect. Particle size represents a critical determinant: nanoparticles in the 10–200 nm range are generally considered optimal for exploiting the EPR effect; those smaller than this range are rapidly cleared by the kidneys, whereas larger particles struggle to extravasate through tumor vascular gaps. Carrier-free nanomedicines typically exhibit particle sizes within the 50–200 nm window, thus theoretically satisfying the size requirements for EPR-mediated accumulation. However, as clinical translation advances, the universality and reliability of the EPR effect have come under increasing scrutiny. Although increased tumor accumulation is frequently attributed simplistically to the EPR effect, substantial heterogeneity has been observed across tumor types, preclinical animal models, and human patients, necessitating a more rigorous and nuanced evaluation. Specifically, murine subcutaneous xenograft tumors are commonly established in the loose connective tissue of the dorsal subcutaneous space. This anatomical location lacks dense extracellular matrix constraints, and the associated neovasculature is immature, featuring abnormally enlarged endothelial gaps and consequently heightened vascular permeability. Concurrently, the interstitial fluid pressure remains relatively low, favoring passive targeting and accumulation of nanomedicines. In contrast, human tumors exhibit highly variable vascular permeability, modulated by aberrant angiogenesis, elevated interstitial fluid pressure, and complex microenvironmental differences. As a result, the actual tumor accumulation of nanomedicines in clinical settings often falls significantly short of preclinical expectations. In summary, the pronounced EPR effect routinely observed in animal models frequently fails to translate to human patients, posing a major translational bottleneck for nanomedicine delivery strategies predicated on this phenomenon. To overcome the substantial limitations of the EPR effect in clinical application, future research must adopt an integrated, multidimensional approach centered on three core pillars: refinement of preclinical models, innovation in delivery strategies, and advancement of real-time, quantitative monitoring technologies.

**Reproducibility and clinical translatability.** The issues of reproducibility and clinical translatability remain inadequately addressed and warrant more rigorous, systematic investigation. First, scalable manufacturing processes for carrier-free nanomedicines have yet to be established through comprehensive, process-oriented studies. Although such formulations are routinely prepared at the laboratory scale, critical engineering challenges associated with scale-up to production-relevant quantities (grams to kilograms) remain unresolved. Specifically, the lack of unified, well-defined process control parameters and quality evaluation criteria results in substantial variability in key physicochemical attributes, such as particle size distribution, particle concentration, encapsulation efficiency, zeta potential, and structural integrity, even across different laboratories or between batches produced on the same manufacturing line. This variability directly compromises experimental reproducibility and batch-to-batch consistency, thereby significantly increasing the complexity and uncertainty associated with subsequent clinical evaluation and regulatory approval. Consequently, systematic identification and characterization of critical parameters governing the preparation and quality control of carrier-free nanomedicines, such as the critical aggregation concentration and the assembly–disassembly equilibrium constant, are urgently required. Establishing a standardized parameter framework specifically tailored to this class of formulations has thus become an essential prerequisite for advancing their clinical translation. Furthermore, the absence of protective carrier materials renders the self-assembly behavior of active pharmaceutical ingredients inherently sensitive to process-induced perturbations during scale-up, thereby introducing additional technical complexities. Second, the exploration of administration routes remains unduly narrow. Current research is overwhelmingly focused on intravenous delivery, whereas alternative routes, including oral and topical administration, have received comparatively limited attention. Given the notoriously low oral bioavailability of flavonols, it remains unclear whether carrier-free nanomedicines can meaningfully enhance their intestinal absorption, and how the structural integrity and colloidal stability of these dietary-component-based carrier-free nanomedicines are preserved under the dynamic and highly variable physicochemical conditions of the gastrointestinal tract.

**Elucidating the in vivo fate and action mechanisms.** Although some progresses have been made, it is difficult, to date, to describe clearly the in vivo fate and action mechanisms of such dietary-component-based nanoplatforms, among which the main questions include (i) the spatiotemporal profile of drug release; (ii) the identity and nature of biomolecular interactions with nanomedicines; and (iii) the feasibility of real-time, unambiguous discrimination between intact nanomedicines and released drugs in vivo. To address attrition in clinical development, we need a more sophisticated map of the dietary-component-based nanomedicine delivery itinerary, from systemic dosing to tumor accumulation, cellular internalization, and accurate subcellular location, and a new generation of targeting tools to facilitate efficient tumor penetration, high tumor retention, and accurate subcellular payload delivery. Characterizing the complete spatiotemporal fate of dietary-component-based nanomedicine is not an easy task because of its size (sub-100 nm), dynamic assembly/disassembly, and complicated biology, making it more complicated without proper monitoring strategies. Currently, molecular imaging can monitor the moving tumor landscape in real time during nanomedicine therapy. By labeling nanomedicines or their targeting ligands on the surface of nanomedicines with environment-responsive fluorophores, such as those based on Förster resonance energy transfer, aggregation-induced emission, or aggregation-caused quenching mechanisms, we can quantify receptor expression levels, track treatment-induced changes in receptor density, and visualize immune cell infiltration (e.g., CD8^+^ T cells and macrophage polarization) within the TME. Hence, this type of imaging can also be used to track the change in receptor density because of its interaction with the drug over time, an important parameter that clinicians can monitor at a relatively high resolution. Simultaneously, imaging informs on the nanoscale the payload localization and the trace of its uptake and dissemination from vascular extravasation and interstitial penetration to intracellular uptake and subcellular fate. This multi-parameter data guide patient stratification based on tumor receptor profiles and the immune context, and inform therapeutic optimization via adaptive dosing, sequencing, and combination. Imaging-derived pharmacokinetics and biodistribution characterize intra- and inter-patient heterogeneity, which can guide dose selection, scheduling, and patient selection. The existing studies are preliminary to draw firm conclusions, and systematic in-depth mechanistic investigations are urgently required. Meanwhile, current research predominantly focuses on their fabrication and in vitro pharmacological efficacy, with a notable paucity of mechanistic studies investigating in vivo interactions with gut microbiota. And yet, the in vivo fate of carrier-free nanomedicines is governed by a tripartite interplay among the gut microbiota, host metabolic enzymes, and intrinsic nanomedicines properties. First, the gut microbiota directly metabolizes nanomedicine constituents via enzymatic transformation, leading to metabolic activation (enhanced efficacy), inactivation (reduced efficacy), or bioactivation into toxic metabolites (compromised safety). Second, microbial metabolites indirectly modulate host drug-metabolizing enzymes (e.g., CYP450 isoforms) and transporters through epigenetic regulation and nuclear receptor activation, thereby altering the rates of Phase I and Phase II metabolism as well as the efficiency of transmembrane transport of nanoparticles. Third, nanomedicines exert bidirectional regulatory effects on the gut microbiota (including selective promotion of beneficial bacteria, suppression of pathobionts, and modulation of microbial metabolic output), thereby establishing feedback loops that may sustain, diminish, or improve their pharmacokinetic profiles. Fourth, interindividual variability in nanomedicine pharmacokinetics arises from the synergistic interaction between host genetic polymorphisms in metabolic enzyme genes and individual-specific microbiota compositional and functional features. Future investigations should integrate multi-omics data, including metagenomics, metabolomics, transcriptomics, proteomics, and pharmacokinetics, to construct comprehensive, multidimensional interaction network models. Coupling multi-omics integration [181] with artificial intelligence [182,183], thus enabled analytical approaches, will significantly deepen our mechanistic understanding of the gut microbiota–carrier-free nanomedicine crosstalk.

Carrier-free nanomedicines, while circumventing the rate-limiting step of developing carrier materials into pharmaceutical excipients, still face multifaceted challenges in their in vivo behavior, including the stability of self-assembled structures, drug release kinetics, and identification of metabolites. These complexities impede progress in fundamental research, process optimization, and cost control, thereby necessitating rigorous validation of their clinical translatability. Moreover, it remains to be experimentally confirmed whether carrier-free nanomedicines can be selectively converted into pharmacologically active forms at target sites while maintaining pharmacological inertness in healthy organs and tissues. On the one hand, certain studies directly extrapolate “significant proliferation inhibition” observed in in vitro cellular assays to infer superior in vivo therapeutic efficacy without subsequent in vivo validation. On the other hand, some reports claim “broad clinical application prospects” based solely on antitumor efficacy observed in a single animal model, overlooking interspecies physiological differences and the numerous hurdles inherent to clinical translation. Looking ahead, incorporating patient-derived tumor organoids into the pharmacological evaluation of carrier-free nanomedicines holds promise for more accurately recapitulating the impact of individualized tumor microenvironments on nanodrug delivery and therapeutic response, thereby enhancing the predictive value of in vitro data for in vivo outcomes. Concurrently, integrating multimodal imaging techniques, including fluorescence imaging, photoacoustic imaging, and magnetic resonance imaging, with carrier-free nanomedicine evaluation enables quantitative assessment of critical parameters such as in vivo self-assembly efficiency, tumor accumulation kinetics, and spatiotemporal drug release profiles. This shift from inference-based judgment to direct, quantitative observation will elevate the evaluation of “potential advantages” from speculative interpretation to evidence-driven characterization.

## Figures and Tables

**Table 1:** Multidimensional comparative analysis of carrier-free nanomedicines and traditional carrier-based nanomedicines.

Comparison Dimensions	Carrier-Free Nanomedicine	Traditional Carrier-Based Nanomedicine	Key Difference Analysis
Drug loading capacity	50–90%, with some systems approaching 100%.	Usually below 10%.	Drug loading capacity in the carrier-free system is increased by more than 5 to 9 times, significantly reducing the number of excipients used.
Vector toxicity	No carrier-related toxicity, avoiding the accumulation of carrier materials in the body and associated long-term toxic risks.	Toxicity or immunogenic responses induced by carrier materials (such as polymers, liposomes, metal nanoparticles).	Carrier-free systems offer structural advantages in terms of safety, making them particularly suitable for long-term or repeated dosing scenarios.
Complexity of the preparation process	Relatively simple, primarily driven by molecular self-assembly, π-π stacking, or hydrophobic interactions to form nanostructures, with few steps and mild conditions.	Complex, involving multiple steps such as carrier material synthesis, drug loading, and surface modification, making it difficult to control reproducibility between batches.	The carrier-free system has a short process chain, which facilitates scale-up production and engineering translation.
Quality control difficulty	Challenging: The chemotherapeutic drug has a well-defined molecular structure, but the particle size distribution, morphology stability, and drug release behavior of the assembled system require precise control.	Mature: Well-established standards for nanoparticle characterization and quality evaluation (e.g., particle size, zeta potential, encapsulation efficiency, release profile, etc.) are already in place.	The traditional system has a more mature quality control standard framework, whereas the carrier-free system requires establishing new quality control metrics specifically for supramolecular assemblies.
Cost-effectiveness	High: Eliminates the synthesis and purification steps of carrier materials, reduces raw material costs, increases production efficiency, and significantly lowers unit dosage cost.	Lower: High carrier material costs (e.g., customized polymers, targeting lipids), significant drug loss during preparation, leading to increased overall cost.	Carrier-free systems offer significant economic advantages in industrial cost control.
Therapeutic index improvement	Significant improvement: High drug loading enables high intratumoral drug concentrations, combined with responsive release of drugs in the tumor microenvironment (e.g., high GSH, low pH), enhancing therapeutic efficacy while reducing toxicity.	Limited enhancement: Due to low drug loading capacity, higher doses are required to achieve effective concentrations, which may lead to carrier-related toxic side effects.	Prodrug design without a carrier demonstrates superior performance in enhancing efficacy and reducing toxicity, thereby broadening the therapeutic window.
Applicable scenarios	Suitable for delivery of chemotherapeutic drugs (e.g., paclitaxel); particularly suitable for treating tumors requiring high-dose local exposure, combination therapy, and overcoming multidrug resistance.	Suitable for systemic drug delivery, requiring long-term stability, targeted delivery (ligand modification), and controlled release for chronic disease treatments (e.g., nucleic acid and protein drug delivery).	The two platforms complement each other: the carrier-free system focuses on optimizing chemotherapy for “high efficiency and low toxicity”, while traditional carriers are geared toward complex delivery needs involving “multifunctional integration”.

**Table 2:** Representative paradigms of dietary polyphenol-based, tumor microenvironment-responsive carrier-free nanoplatforms for synergistically enhanced tumor-targeted therapy.

Dietary Components	Therapeutic Agents	Size [nm]	Therapy Methods	Results	Refs.
Curcumin	CI-NPs	54.6	Chemo-photothermal therapy	Inhibits tumor growth, suppresses metastasis, and improves safety.	[51]
	Cur@IR783 NC	28.4	Chemo-photothermal therapy	Accumulates at the tumor site, enhances antitumor efficacy.	[52]
	PBSN38-CUR	128.0	Chemo-immunotherapy	Activates the cGAS–STING pathway through amplification of DNA damage.	[53]
Isoliquiritigenin	ISL@RLP-Fe	120.9	Chemo-chemodynamic-immunotherapy	Significantly reduces tumor mass and inhibits cancer cell migration.	[54]
Mangiferin	MP NPs	~68	Chemotherapy	Overcomes drug resistance via a multi-pronged strategy: modulating inflammatory TME, inducing ferroptosis, and enabling deep tumor penetration.	[55]
EGCG	Fe-EGCG@RSL3	~200	Chemotherapy, immunotherapy	Boosts anti-PD-1 immunotherapy via ferroptosis induction and immunomodulation.	[56]
Erianin and curcumin	CUR@CPT-SS-ERI NPs	108.0	Chemotherapy	Mitigates multidrug resistance in lung cancer while enhancing efficiency.	[57]
Rosmarinic acid	Fe–RA	~200	Ferroptosis-based chemotherapy	Fe–phenolic network synergizes with ferroptosis to enhance nanotherapy.	[58]
Capsaicin	Ir-Gem@Cap	86.5	Chemotherapy and photodynamic therapy	Chemo-, photo-, and anti-cluster therapies jointly suppress tumor growth and metastasis.	[59]
Capsaicin and curcumin	Cap-p@Cur	~460	Chemotherapy and photodynamic therapy	Enhances cytotoxicity, further amplified by light irradiation.	[60]
Quercetin	HDNP	159.6	Chemotherapy, immunotherapy	Synergistic ferroptosis and immunotherapy to overcome tumor resistance and metastasis.	[61]
	QN NP	250.0	Chemotherapy, immunotherapy	Disrupts cancer-associated fibroblast barriers and alleviates immunosuppression to potentiate immunotherapy.	[62]
	AFQ@HA	98.1	Chemo-, photothermal, and chemodynamic therapy	Achieves enhanced antitumor effect via CD44-mediated tumor targeting—passive and active—in response to the tumor microenvironment, with excellent efficacy.	[63]
Procyanidin	PFGH	91.3	Chemotherapy and photothermal therapy	Inhibits TMEM16A–EGFR mutual activation for photothermally enhanced breast cancer therapy.	[64]
Baicalein	BC NPs	~80	Photoimmunotherapy	Improves breast cancer treatment for resistant or advanced cases.	[65]
Chlorogenic acid	CCS-NDs	198.0	Ferroptosis-based chemotherapy	Triggers massive lipid peroxides (LPO) accumulation and ferroptosis in tumor cells.	[66]
Galangin	FSSN/GA NAs	~110	Chemosensitizers improved chemotherapy	Expands the therapeutic window of SN38 by integrating prodrug activation and chemosensitization strategies.	[67]

## Data Availability

No data were used for the research described in the article.

## References

[1] Schapira L., Duffy C.M. (2025). Cancer treatment and survivorship statistics, 2025: An urgent call to optimize health after cancer. CA-Cancer J. Clin..

[2] Schaue D., McBride W.H. (2015). Opportunities and challenges of radiotherapy for treating cancer. Nat. Rev. Clin. Oncol..

[3] Chen Y., Chen S., Chen K., Ji L., Cui S. (2024). Magnolol and 5-fluorouracil synergy inhibition of metastasis of cervical cancer cells by targeting PI3K/AKT/mTOR and EMT pathways. Chin. Herb. Med..

[4] Yang L., Wang Z. (2026). Podophyllotoxin-based intelligent nanoplatforms for augmented tumor therapy: Strategies, challenges, and perspectives. Mater. Chem. Front..

[5] Yang L., Wang Z. (2025). The ballet of natural-product: Carrier-free “triadic” drug delivery platforms for enhanced tumor treatment. J. Funct. Biomater..

[6] Yang L.-Y., Wang Y., Wang X., Wang R., Wang Y.-H., Wang K.-D., Wang Z.-L. (2027). Natural product–based multifunctional carrier-free nanoplatforms for reinvigorating antitumor immunity. Tradit. Med. Res..

[7] Wang Z., Song X., Sun M., Zhang R., Yang L. (2025). Bench-to-bedside translation of podophyllotoxin-based nanomedicines for cancer treatment: Utopias and reality?. Microstructures.

[8] Slingerland N., Runderkamp E., Thommen D.S. (2026). Immune niches in cancer. Annu. Rev. Immunol..

[9] Hanahan D. (2026). Hallmarks of cancer—Then and now, and beyond. Cell.

[10] Xu B. (2025). Redefining the future of cancer care: Intelligent oncology unveiled. Intell. Oncol..

[11] Shu Y., Ling J., Wu Z., Le G., Wu J., Xia G., Li W. (2026). Analysis of key technologies and intelligent development trends for tumor surgery navigation platforms. Intell. Oncol..

[12] De Ruysscher D., Damen P.J., Hendriks L.E., Saw S.P. (2026). Radiotherapy has a role to play in patients with EGFR-mutated non-small-cell lung cancer in first-line treatment. Lancet Oncol..

[13] Wang Z., Sun X., Sun M., Wang C., Yang L. (2025). Game changers: Blockbuster small-molecule drugs approved by the FDA in 2024. Pharmaceuticals.

[14] Bag J., Hazra R., Mahish R., Dey A.K., Sukul D., K A.K.S., De S. (2026). A mini-review on the multimodal applications of engineered magnetic nanoparticles in diagnosis and therapy. Nano TransMed.

[15] Falzone L., Bordonaro R., Libra M. (2023). SnapShot: Cancer chemotherapy. Cell.

[16] Yang L.Y., Lei S.Z., Xu W.J., Lai Y.X., Zhang Y.Y., Wang Y., Wang Z.L. (2025). Rising above: Exploring the therapeutic potential of natural product-based compounds in human cancer treatment. Tradit. Med. Res..

[17] Kou X., Wu X., He T., Bai L., Li X., Luo R., Xu Y., Wang Y., Shen M., Gong C. (2026). Tumor-activated dual metabolic nanomodulator turns over tolerogenic tumor control and unleashes systemic immune responses. J. Nanobiotechnol..

[18] Muñoz-Rojas A.R., Mathis D. (2021). Tissue regulatory T cells: Regulatory chameleons. Nat. Rev. Immunol..

[19] Yuan T., Liu J., Zhang C., Lv X., Tan G., Xue L., Zhang E., Liang H., Huang Z. (2026). Tumor-induced rewiring of splenic niches: From immune organ to cancer accomplice. Int. J. Biol. Sci..

[20] Yu Y., Yao X., Wang Q., Yang M., Li R., Qin J., Zhuang J., Sun C. (2026). T cell exhaustion in cancer immunotherapy: Heterogeneity, mechanisms, and therapeutic opportunities. Adv. Sci..

[21] Darragh L.B., Karam S.D. (2026). Radiation as an immune modulator: Mechanisms and implications for combination with immunotherapy. Nat. Rev. Cancer.

[22] Luo G., Jiang X., Hu C., Li L., Yan L., Xiao G., Duo Y., Zhang X. (2026). Artificial intelligence-powered nanomedicine. Chem. Soc. Rev..

[23] Wang W., Mu S., Zhang J., Shan G., Li X., Wang R., Guo T., He X. (2026). Trojan horse strategy: How biomimetic nanomedicine remodels the tumor microenvironment. Adv. Sci..

[24] Yang L., Wang Y., Ye X., Liu Q., Qu D., Chen Y. (2024). Traditional Chinese medicine-based drug delivery systems for anti-tumor therapies. Chin. J. Nat. Med..

[25] Esmaeilpour D., Hamblin M.R., Cheng J., Khosravi A., Liu J., Zarepour A., Zarrabi A., Sillanpää M., Zare E.N., Shen J. (2026). Artificial intelligence driven protein design and sustainable nanomedicine for advanced theranostics. Bio Mat..

[26] Wang Z., Yang L. (2024). Natural-product-based, carrier-free, noncovalent nanoparticles for tumor chemo-photodynamic combination therapy. Pharmacol. Res..

[27] Wang Z., Yang L. (2020). Turning the tide: Natural products and natural-product-inspired chemicals as potential counters to SARS-CoV-2 infection. Front. Pharmacol..

[28] Wang X., Miao Y.H., Zhao X.M., Liu X., Hu Y.W., Deng D.W. (2024). Perspectives on organ-on-a-chip technology for natural products evaluation. Food Med. Homol..

[29] Wang Z., Song X., Xu W., Lei S., Zhang H., Yang L. (2023). Stand up to stand out: Natural dietary polyphenols curcumin, resveratrol, and gossypol as potential therapeutic candidates against severe acute respiratory syndrome coronavirus 2 infection. Nutrients.

[30] Liu T., Yang L., Dong C., Qi D., Ji B., Gao Q. (2024). Spectrum-effect relationship between components and antitumor activity of Lonicerae Japonicae Flos based on orthogonal partial least squares regression. Sci. Tradit. Chin. Med..

[31] Wang Z. (2019). Advances in the asymmetric total synthesis of natural products using chiral secondary amine catalyzed reactions of α,β-unsaturated aldehydes. Molecules.

[32] Wang Z., Wang N., Yang L., Song X.Q. (2022). Bioactive natural products in COVID-19 therapy. Front. Pharmacol..

[33] Wang Z., Yang L., Yang X., Zhang X. (2014). Advances in the chemical synthesis of artemisinin. Synth. Commun..

[34] Wang Z., Yang L. (2023). The therapeutic potential of natural dietary flavonoids against SARS-CoV-2 infection. Nutrients.

[35] Liu Y., Meng J., Liu J., Zhang F., An C., Yang J. (2025). NLRP3 inflammasome in acute lung injury: Cumulative evidence for traditional Chinese medicine and natural products. Chin. Herb. Med..

[36] Yang L., Wang Z. (2023). Bench-to-bedside: Innovation of small molecule anti-SARS-CoV-2 drugs in China. Eur. J. Med. Chem..

[37] Mohanasundaram P., Antoneyraj M.S. (2025). A systematic review of neem flower (*Azadirachta indica*): A promising source of bioactive compounds with pharmacological and immunomodulating properties. Tradit. Med. Res..

[38] Takemoto M., Delghandi S., Abo M., Yurimoto K., Odagi M., Singh V., Wang J., Nakagawa R., Sato S.I., Takemoto Y. (2025). Covalent plant natural product that potentiates antitumor immunity. J. Am. Chem. Soc..

[39] Feng W., Fang D., Tang F., Gao J., Chen F., Li Z., Duan C., Zhang Y., Yu M., Wang P. (2025). Icariside II attenuates isoproterenol-induced myocardial ischemia by regulating NLRP3/Caspase-1 axis. Sci. Tradit. Chin. Med..

[40] Zhao Y., Deng S., Cao D., Lin C., Xu M., Wang J., Luo L., Liu S., Liu H. (2025). Mechanisms and benefits of phytochemicals as an alternative therapeutic strategy in female cancers. Chin. Herb. Med..

[41] Yu Y.-Y., Fu H.-Q., Du H.-Y., Zhang J., Chen K.-Y., Zhao Y.-X., Gao Y., Li M.-H. (2025). Overview of research on the application of medicine food homologous bioactive ingredients to functional constipation. Food Med. Homol..

[42] Zhao Z., Ma Y., Wang S., Zhou M., Tian Y., Yang L., Gu J., Tan R. (2025). Natural compounds from traditional Chinese medicine regulating stem cell fate: Identification and therapeutic potential. Chin. Herb. Med..

[43] Yang L., Wang Z. (2021). Natural products, alone or in combination with FDA-approved drugs, to treat COVID-19 and lung cancer. Biomedicines.

[44] Wu W., Mi Y., Meng Q., Li N., Li W., Wang P., Hou Y. (2025). Natural polyphenols as novel interventions for aging and age-related diseases: Exploring efficacy, mechanisms of action and implications for future research. Chin. Herb. Med..

[45] Guo X., Zhang L., Wu Y., Luan X., Ge G. (2026). Integrating multi-omics technologies in traditional Chinese medicine: Novel insights into cancer therapy. World J. Integr. Tradit. West. Med..

[46] Wang Z., Yang L., Li Y., Song S., Qu J., He R., Ren S., Gong P. (2022). An activatable, carrier-free, triple-combination nanomedicine for ALK/EGFR-mutant non-small cell lung cancer highly permeable targeted chemotherapy. N. J. Chem..

[47] Wang Y., Huang R., Feng S., Mo R. (2025). Advances in nanocarriers for targeted drug delivery and controlled drug release. Chin. J. Nat. Med..

[48] Yang L., Song X., Wang Z. (2026). TRAIL of Hope: Bioactive dietary component-driven carrier-free nanoplatforms for synergistically enhanced tumor multimodal therapy. Int. J. Nanomed..

[49] Liu L.H., Zhang X.Z. (2022). Carrier-free nanomedicines for cancer treatment. Prog. Mater. Sci..

[50] Fang F., Chen X. (2024). Carrier-free nanodrugs: From bench to bedside. ACS Nano.

[51] Chen Y., Ran Y., Rao Z., Wang Q., Wang J., Liu Z., Zhou Q., Lei X., Xu Z., Ming J. (2024). Self-assembled chemo/photothermal nanoplatform for enhanced cancer therapy: Sensitizing photothermal ablation and reprogramming inflammatory microenvironment. ACS Mater. Lett..

[52] Zheng M., Zhang J., Deng C., Chen L., Zhang H., Xin J., Aras O., Zhou M., An F., Ren Y. (2024). The collaborated assembly of hydrophobic curcumin and hydrophilic cyanine dye into nanocolloid for synergistic chemo-photothermal cancer therapy. Mater. Des..

[53] Xu X., Jin P., Chen Y., Wu B., Fang X., Song Y., Luo J., Wang G., Huang P. (2026). Ultrasound-triggered carrier-free nanoprodrugs activate cGAS-STING pathway to enhance tumor-targeting chemo-immunotherapy. Mater. Today Bio.

[54] Lin Z., Cao R., Guo X., Nie F., Xu J., Guo Y. (2025). Herbal medicine-derived natural product self-assembled nanoparticles: Orchestrating chemo-chemodynamic-immunotherapy for tumor combination therapy. Adv. Healthc. Mater..

[55] Zheng Y., Xu R., Chen T., Wang Y., Chen X., Chen H., Gao Y. (2025). Four-pronged reversal of chemotherapy resistance by mangiferin amphiphile. J. Control. Release.

[56] Zhang C., Liu S., Zhang J., Lu J., Chen Z., Pan B., Liu C., Huang M., Zhan H., Wang H. (2025). A multifunctional Fe-EGCG@ RSL3 nanomedicine synergizes ferroptosis induction and tumor microenvironment remodeling for enhanced bladder cancer immunotherapy. Research.

[57] Yang L., Zhang Y., Lai Y., Xu W., Lei S., Wang Z. (2025). A computer-aided, heterodimer-based “triadic” carrier-free drug delivery platform to mitigate multidrug resistance in lung cancer and enhance efficiency. J. Colloid Interface Sci..

[58] Chen Y., Yang X., Li H., Wu X., Wu W., Chen J., Wu A., Wang X. (2024). Self-assembled Fe-phenolic acid network synergizes with ferroptosis to enhance tumor nanotherapy. Small.

[59] Shan J., Liu C., Yuan L., Jiang L., Sun Y. (2025). “Three-birds with one stone” self-assembled carrier-free drug delivery system for efficient inhibition of breast cancer growth and metastasis. Colloid Surf. B.

[60] Liu D., Zou Z., Hu X., Huang S., Huang T., Gu J., Zhu W., Liu M., Huang S., Zhang X. (2025). Self-assembly of phosphorylated capsaicin and curcumin for treatment of triple-negative breast cancer via combination of chemotherapy and photodynamic therapy. Colloid Surf. A.

[61] Zhang N., Wang Z., Li G., Zhang M., Liu Q., Cai C., Shang Y., Zhu H., An H., Ren S. (2024). A carrier-free metal-phenolic network with enhanced ferroptosis-immunotherapy for overcoming tumor resistance and metastasis. Chem. Eng. J..

[62] Li S., Yue Y., Du J., Zhao W., Liu B., Ma Z., Wang X., Wei Y., Song C., Zhang G. (2026). Carrier-free nanoreshapers disrupt cancer-associated fibroblast barriers and alleviate immunosuppression for synergistically potentiated immunotherapy. J. Control. Release.

[63] Tang H.-X., He Z.-H., Liu C.-G., Zheng X.-K., Zhang Z.-J. (2024). Tumor microenvironment-responsive biodegradable nanomedicine for self-enhanced synergistic chemo-, photothermal, and chemodynamic therapy. ACS Appl. Mater. Interfaces.

[64] Li G., Cai C., Zhang N., Wang Z., Zhang M., Liu Q., Chen S., Shang Y., Zhao C., Ren S. (2025). Carrier-free procyanidin-ferric nanonetworks loading gefitinib to inhibit TMEM16A-EGFR mutual activation loop for photothermally enhanced breast cancer targeted therapy. Mater. Today Bio.

[65] Xiang J., Suo M., Lan J., Ning S., Liu H., Hong W., Liang J., Zheng J., Zhu D., Mo J. (2025). Novel carrier-free nanomedicine for regulating macrophage phenotype to amplify anti-tumor photoimmunotherapy. Small.

[66] Zhang H., Shan G., Liu M., Sun Q., Yang T., Peng R., Li X., Mei Y., He X., Qiao L. (2025). Harnessing ROS amplification and GSH depletion using a carrier-free nanodrug to enhance ferroptosis-based cancer therapy. Small.

[67] Wu C., Wang Y., Lin Z., Yan W., Chen Q., Zhang S., Sun J., He Z., Luo C. (2025). Galangin nano-cocktail balances prodrug activation and antitumor response for enhanced cancer treatment. Small.

[68] Peng S., Xiao F., Chen M., Gao H. (2022). Tumor-microenvironment-responsive nanomedicine for enhanced cancer immunotherapy. Adv. Sci..

[69] Esfanddarani H.M., Panigrahi M. (2024). Phytosynthesis of transition (Ni, Fe, Co, Cr, and Mn) metals and their oxide nanoparticles for biomedical applications: A review. J. Mater. Sci..

[70] Fu Q., Wei C., Wang M. (2024). Transition-metal-based nanozymes: Synthesis, mechanisms of therapeutic action, and applications in cancer treatment. ACS Nano.

[71] Li S.L., Jiang P., Jiang F.L., Liu Y. (2021). Recent advances in nanomaterial-based nanoplatforms for chemodynamic cancer therapy. Adv. Funct. Mater..

[72] Ma S., Li D., Jia X., Xu W., Ding G., He J., Wang J. (2024). Homologous tumor targeting molybdenum-doped prussian blue for enhancing immunotherapy via PTT/CDT and remodeled tumor immune microenvironment. Adv. Funct. Mater..

[73] Mustafa A.M., El-Shiekh R.A., Esmail M.M., Hassan E., Senna M.M., Ebid N., Elgindy A.M. (2025). Surveying the therapeutic potentials of isoliquiritigenin (ISL): A comprehensive review. Chem. Biodivers..

[74] Chen Z., Ding W., Yang X., Lu T., Liu Y. (2024). Isoliquiritigenin, a potential therapeutic agent for treatment of inflammation-associated diseases. J. Ethnopharmacol..

[75] Huang Y., Sun J., Li S., Shi Y., Yu L., Wu A., Wang X. (2024). Isoliquiritigenin mitigates intervertebral disc degeneration induced by oxidative stress and mitochondrial impairment through a PPARγ-dependent pathway. Free Radic. Biol. Med..

[76] Shi F., Du M., Wang Q., Adu-Frimpong M., Li C., Zhang X., Ji H., Toreniyazov E., Cao X., Wang Q. (2024). Isoliquiritigenin containing PH sensitive micelles for enhanced anti-colitis activity. J. Pharm. Sci..

[77] Li C., Huang Y., Liu Y., Li Z., Deng Y., Ren H., Zhao D., Han L., Sun J. (2025). Self-assembly of Zein-based nanoparticles for oral isoliquiritigenin delivery against Salmonella infections. Innov. Food Sci. Emerg..

[78] Peng S., Gu P., Mao N., Yu L., Zhu T., He J., Yang Y., Liu Z., Wang D. (2024). Structural characterization and in vitro anti-inflammatory activity of polysaccharides isolated from the fruits of Rosa laevigata. Int. J. Mol. Sci..

[79] Quan X.X., Huang Y.Y., Chen L., Yuan J.Q. (2022). Traditional uses, phytochemical, pharmacology, quality control and modern applications of two important Chinese medicines from Rosa laevigata Michx: A review. Front. Pharmacol..

[80] Guo X., Nie F., Jiang H., Che S., Liao H.-B., Xu J., Guo Y. (2025). A bioactive polysaccharide derived from Rosa laevigata fruits: Structural properties, antitumor efficacy, and potential mechanisms. Int. J. Biol. Macromol..

[81] Oyalo J.O., Gikundi E.N., Ndiege M.A., Aduol K.O. (2025). Bioactive polysaccharides and their potential health benefits in reducing the risks of metabolic syndrome: A review. Food Biomacromol.

[82] Gao Q., Zhan Y., Sun L., Zhu W. (2023). Cancer stem cells and the tumor microenvironment in tumor drug resistance. Stem Cell Rev..

[83] Xu Q., Lan X., Lin H., Xi Q., Wang M., Quan X., Yao G., Yu Z., Wang Y., Yu M. (2023). Tumor microenvironment-regulating nanomedicine design to fight multi-drug resistant tumors. WIREs Nanomed. Nanobiotechnol..

[84] Kaemmerer E., Loessner D., Avery V.M. (2021). Addressing the tumour microenvironment in early drug discovery: A strategy to overcome drug resistance and identify novel targets for cancer therapy. Drug Discov. Today.

[85] Iqbal H., Inam-Ur-Raheem M., Munir S., Rabail R., Kafeel S., Shahid A., Khaneghah A.M., Aadil R.M. (2024). Therapeutic potential of mangiferin in cancer: Unveiling regulatory pathways, mechanisms of action, and bioavailability enhancements–an updated review. Food Sci. Nutr..

[86] Zivković J., Kumar K.A., Rushendran R., Ilango K., Fahmy N.M., El-Nashar H.A.S., El-Shazly M., Ezzat S.M., Melgar-Lalanne G., Romero-Montero A. (2024). Pharmacological properties of mangiferin: Bioavailability, mechanisms of action and clinical perspectives. Naunyn-Schmiedeberg’s Arch. Pharmacol..

[87] Mei S., Perumal M., Battino M., Kitts D.D., Xiao J., Ma H., Chen X. (2023). Mangiferin: A review of dietary sources, absorption, metabolism, bioavailability, and safety. Crit. Rev. Food Sci..

[88] Nestmann E.R., Alluri V.K., Dodda S., Davis B.A. (2019). Toxicological studies on the botanical supplement LI12542F6 containing extracts of Sphaeranthus indicus flower heads and Mangifera indica (mango tree) bark. Food Sci. Nutr..

[89] Irshad N., Naeem H., Shahbaz M., Imran M., Mujtaba A., Hussain M., Al Abdulmonem W., Alsagaby S.A., Yehuala T.F., Abdelgawad M.A. (2024). Mangiferin: An effective agent against human malignancies. Food Sci. Nutr..

[90] Sarfraz M., Khan A., Batiha G.E.-S., Akhtar M.F., Saleem A., Ajiboye B.O., Kamal M., Ali A., Alotaibi N.M., Aaghaz S. (2023). Nanotechnology-based drug delivery approaches of mangiferin: Promises, reality and challenges in cancer chemotherapy. Cancers.

[91] Tao Y., Tian X., Luo J., Zhu H., Chu Y., Pei L. (2023). Mangiferin inhibits chronic stress-induced tumor growth in colorectal liver metastases via WAVE2 signaling pathway. Heliyon.

[92] Li L., Dong Y., Liu X., Wang M. (2023). Mangiferin for the management of liver diseases: A review. Foods.

[93] Cheng S., Wang Y., Tong T., Liu C., Qian J., Zong Q., Wang D., Li K., Wu X., Yang J. (2025). Mangiferin attenuates ang II-induced abdominal aortic aneurysm by blocking Lys591-mediated nuclear translocation of STAT3. Phytomedicine.

[94] Deng X., Lin B., Wang F., Xu P., Wang N. (2024). Mangiferin attenuates osteoporosis by inhibiting osteoblastic ferroptosis through Keap1/Nrf2/SLC7A11/GPX4 pathway. Phytomedicine.

[95] Castro-Muñoz R., Cabezas R., Plata-Gryl M. (2024). Mangiferin: A comprehensive review on its extraction, purification and uses in food systems. Adv. Colloid Interface Sci..

[96] Melo-Betances E., Rodríguez-Bautista C.C., Núñez-Sellés A.J. (2025). Synthesis of mangiferin derivatives, complexes, and carriers as potential therapeutic candidates for cancer treatment: An update. Front. Pharmacol..

[97] Sandbhor P., Palkar P., Bhat S., John G., Goda J.S. (2024). Nanomedicine as a multimodal therapeutic paradigm against cancer: On the way forward in advancing precision therapy. Nanoscale.

[98] Yu S., Zhou Y., Sun Y., Wu S., Xu T., Chang Y., Bi S., Jiang L., Zhu J. (2021). Endogenous mRNA triggered DNA-Au nanomachine for in situ imaging and targeted multimodal synergistic cancer therapy. Angew. Chem. Int. Ed..

[99] Mishra S.K., Dhadve A.C., Mal A., Reddy B.P.K., Hole A., Chilakapati M.K., Ray P., Srivastava R., De A. (2022). Photothermal therapy (PTT) is an effective treatment measure against solid tumors which fails to respond conventional chemo/radiation therapies in clinic. Biomater. Adv..

[100] Li L., Zhang X., Zhou J., Zhang L., Xue J., Tao W. (2022). Non-invasive thermal therapy for tissue engineering and regenerative medicine. Small.

[101] Gao G., Sun X., Liang G. (2021). Nanoagent-promoted mild-temperature photothermal therapy for cancer treatment. Adv. Funct. Mater..

[102] Xiang H., Zhao L., Yu L., Chen H., Wei C., Chen Y., Zhao Y. (2021). Self-assembled organic nanomedicine enables ultrastable photo-to-heat converting theranostics in the second near-infrared biowindow. Nat. Commun..

[103] Wang Y., Ma L., Hu J., Liu Y., Zhao B., Gao Y. (2026). Carrier-free self-assembled nanodrugs in cancer therapy: A systematic scoping review and evidence map of molecular architecture, drug resistance, and translation-relevant reporting. Front. Pharmacol..

[104] Barreca D., Trombetta D., Smeriglio A., Mandalari G., Romeo O., Felice M.R., Gattuso G., Nabavi S.M. (2021). Food flavonols: Nutraceuticals with complex health benefits and functionalities. Trends Food Sci. Technol..

[105] Gupta E., Mishra P. (2021). Functional food with some health benefits, so called superfood: A review. Curr. Nutr. Food Sci..

[106] Premji T.P., Dash B.S., Das S., Chen J.P. (2024). Functionalized nanomaterials for inhibiting ATP-dependent heat shock proteins in cancer photothermal/photodynamic therapy and combination therapy. Nanomaterials.

[107] Peng J., Li S., Ti H. (2024). Sensitize tumor immunotherapy: Immunogenic cell death inducing nanosystems. Int. J. Nanomed..

[108] Sun H., Zhang Q. (2024). Recent advances in perylene diimides (PDI)-based small molecules used for emission and photothermal conversion. ChemPhotoChem.

[109] Wu J., Hu X., Chang Y., Sun Z., Zhou M., Song S., Chen Z., Wu Y. (2025). pH/reduction/light-responsive nanodrug based on perylene diimide polyprodrug for combined chemotherapy and photothermal therapy. ACS Appl. Nano Mater..

[110] Fan M., Zheng X., Cheng W., Wei H., Li P., Ji C., Yin M. (2024). Reinforcing carrier-free photothermal nanodrugs through flavonol-driven assembly. Adv. Funct. Mater..

[111] van Hoogstraten L.M.C., Vrieling A., van der Heijden A.G., Kogevinas M., Richters A., Kiemeney L.A. (2023). Global trends in the epidemiology of bladder cancer: Challenges for public health and clinical practice. Nat. Rev. Clin. Oncol..

[112] Lopez-Beltran A., Cookson M.S., Guercio B.J., Cheng L. (2024). Advances in diagnosis and treatment of bladder cancer. BMJ.

[113] Zhou Z., Lv Y., Zuo A., Zhu X., Xu Y., Zuo L., Xu H., Liu S., Zhang Y., Weng S. (2025). Interactions between microbiota and innate immunity in tumor microenvironment: Novel insights into cancer progression and immunotherapy. hLife.

[114] Gao W., Wang X., Zhou Y., Wang X., Yu Y. (2022). Autophagy, ferroptosis, pyroptosis, and necroptosis in tumor immunotherapy. Sig Transduct. Target. Ther..

[115] Gong D., Chen M., Wang Y., Shi J., Hou Y. (2022). Role of ferroptosis on tumor progression and immunotherapy. Cell Death Discov..

[116] Ntamo Y., Jack B., Ziqubu K., Mazibuko-Mbeje S.E., Nkambule B.B., Nyambuya T.M., Mabhida S.E., Hanser S., Orlando P., Tiano L. (2024). Epigallocatechin gallate as a nutraceutical to potentially target the metabolic syndrome: Novel insights into therapeutic effects beyond its antioxidant and anti-inflammatory properties. Crit. Rev. Food Sci..

[117] Capasso L., De Masi L., Sirignano C., Maresca V., Basile A., Nebbioso A., Rigano D., Bontempo P. (2025). Epigallocatechin gallate (EGCG): Pharmacological properties, biological activities and therapeutic potential. Molecules.

[118] Krzyzowska M., Janicka M., Chodkowski M., Patrycy M., Obuch-Woszczatyńska O., Tomaszewska E., Ranoszek-Soliwoda K., Celichowski G., Grobelny J. (2023). Epigallocatechin gallate-modified silver nanoparticles show antiviral activity against herpes simplex type 1 and 2. Viruses.

[119] Noman A.M., Sultan M.T., Mazhar A., Baig I., Javaid J., Hussain M., Imran M., Alsagaby S.A., Al Abdulmonem W., Mujtaba A. (2025). Anticancer molecular mechanisms of epigallocatechin gallate: An updated review on clinical trials. Food Sci. Nutr..

[120] Ma C., Xie Y., Huang X., Zhang L., McClements D.J., Zou L., Liu W. (2024). Encapsulation of (-)-epigallocatechin gallate (EGCG) within phospholipid-based nanovesicles using W/O emulsion-transfer methods: Masking bitterness and delaying release of EGCG. Food Chem..

[121] Theobald S., Pfeiffer P., Zuber U., König H. (2008). Influence of epigallocatechin gallate and phenolic compounds from green tea on the growth of Oenococcus oeni. J. Appl. Microbiol..

[122] Cha S.Y., Park S.Y., Lee J.S., Lee K.H., Kim J.H., Fang Y., Shin S.S. (2022). Efficacy of Dendrobium candidum polysaccharide extract as a moisturizer. J. Cosmet. Dermatol..

[123] Lei W., Luo J., Wu K., Chen Q., Hao L., Zhou X., Wang X., Liu C., Zhou H. (2021). Dendrobium candidum extract on the bioactive and fermentation properties of Lactobacillus rhamnosus GG in fermented milk. Food Biosci..

[124] Zheng T., Kan X., Zhang L., Zhang A., Huang X., Zhao C., Wang R., Lu J. (2021). Research progress on chemical constituents and functions of dendrobium candidum. Int. Core J. Eng..

[125] Chen F., Chen Z., Sun H., Zhu J., Wu K., Zhou S., Huang Y. (2022). Dendrobium candidum quality detection in both food and medicine agricultural product: Policy, status, and prospective. Front. Sustain. Food Syst..

[126] Qiao Q., Du Y., Xie L. (2022). Research advances of erianin: Source, production, biological activities and pharmacological properties. Pharmacol. Res. Mod. Chin. Med..

[127] Miao Q., Deng W.-Q., Lyu W.-Y., Sun Z.-T., Fan S.-R., Qi M., Qiu S.-H., Zhu Y.-R., Lin J.-P., Chen M.-F. (2023). Erianin inhibits the growth and metastasis through autophagy-dependent ferroptosis in KRASG13D colorectal cancer. Free Radic. Biol. Med..

[128] Ma L., Li M., Zhang Y., Liu K. (2023). Recent advances of antitumor leading compound erianin: Mechanisms of action and structural modification. Eur. J. Med. Chem..

[129] Zhang X., Hu L., Xu S., Ye C., Chen A. (2021). Erianin: A direct NLRP3 inhibitor with remarkable anti-inflammatory activity. Front. Immunol..

[130] Xie H., Feng S., Farag M.A., Sun P., Shao P. (2021). Synergistic cytotoxicity of erianin, a bisbenzyl in the dietetic Chinese herb Dendrobium against breast cancer cells. Food Chem. Toxicol..

[131] Mansuer M., Zhou L., Wang C., Gao L., Jiang Y. (2024). Erianin induces ferroptosis in GSCs via REST/LRSAM1 mediated SLC40A1 ubiquitination to overcome TMZ resistance. Cell Death Dis..

[132] Zhong Y., Cen Y., Xu L., Li S., Cheng H. (2023). Recent progress in carrier-free nanomedicine for tumor phototherapy. Adv. Healthc. Mater..

[133] Memarzia A., Khazdair M.R., Behrouz S., Gholamnezhad Z., Jafarnezhad M., Saadat S., Boskabady M.H. (2021). Experimental and clinical reports on anti-inflammatory, antioxidant, and immunomodulatory effects of Curcuma longa and curcumin, an updated and comprehensive review. BioFactors.

[134] Lima E.P., Gonçalves O.H., Ames F.Q., Castro-Hoshino L.V., Leimann F.V., Cuman R.K.N., Comar J.F., Bersani-Amado C.A. (2021). Anti-inflammatory and antioxidant activity of nanoencapsulated curcuminoids extracted from Curcuma longa L. in a model of cutaneous inflammation. Inflammation.

[135] Khan M.A., Khan A., Khan A.A., Mirza M.A., Naquvi K.J., Kumar D. (2025). Anti-carcinogenic potential of nano-curcumin “killing cancerous cells”. Curr. Bioact. Comp..

[136] Wang F., Li Y., Jiang H., Li C., Li Z., Qi C., Li Z., Gao Z., Zhang B., Wu J. (2021). Dual-ligand-modified liposomes co-loaded with anti-angiogenic and chemotherapeutic drugs for inhibiting tumor angiogenesis and metastasis. Int. J. Nanomed..

[137] Hu P., Li K., Peng X.-X., Kan Y., Yao T.-J., Wang Z.-Y., Li Z., Liu H.-Y., Cai D. (2023). Curcumin derived from medicinal homologous foods: Its main signals in immunoregulation of oxidative stress, inflammation, and apoptosis. Front. Immunol..

[138] Alqahtani Q.H., Fadda L.M., Alhusaini A.M., Hasan I.H., Ali H.M. (2023). Involvement of Nrf2, JAK and COX pathways in acetaminophen-induced nephropathy: Role of some antioxidants. Saudi Pharm. J..

[139] Bučević Popović V., Karahmet Farhat E., Banjari I., Jeličić Kadić A., Puljak L. (2024). Bioavailability of oral curcumin in systematic reviews: A methodological study. Pharmaceuticals.

[140] Luo Y., Zhu L., Ren Z., Xiao J., Hao E., Lu J., Zhao J., Yao C., Wang Y., Luo H. (2025). Curcumae Rhizoma: An anti-cancer traditional Chinese medicine. Chin. Herb. Med..

[141] Huang S., Xu D., Zhang L., Hao L., Jia Y., Zhang X., Cheng T., Chen J. (2023). Therapeutic effects of curcumin liposomes and nanocrystals on inflammatory osteolysis: In vitro and in vivo comparative study. Pharmacol. Res..

[142] Rostami N., Ghebleh A., Noei H., Rizi Z.S., Moeinzadeh A., Nikzad A., Gomari M.M., Uversky V.N., Tarighi P. (2024). Peptide-functionalized polymeric nanoparticles for delivery of curcumin to cancer cells. J. Drug Deliv. Sci. Tech..

[143] Ma L., Gao H., Cheng C., Cao M., Zou L., Liu W. (2023). Fabrication of emulsions using high loaded curcumin nanosuspension stabilizers: Enhancement of antioxidant activity and concentration of curcumin in micelles. J. Funct. Foods.

[144] Chelimela N., Alavala R.R., Satla S.R. (2024). Curcumin-bioavailability enhancement by prodrug approach and novel formulations. Chem. Biodivers..

[145] Wei H., Jiang D., Yu B., Ni D., Li M., Long Y., Ellison P.A., Siamof C.M., Cheng L., Barnhart T.E. (2023). Nanostructured polyvinylpyrrolidone-curcumin conjugates allowed for kidney-targeted treatment of cisplatin induced acute kidney injury. Bioact. Mater..

[146] Wang X., Wang Y., Luo R., Niu D., Yang L., Wang Z. (2026). Small-Molecule Drugs Approved by the FDA in 2025. Molecules.

[147] Zhang Y., You P., Liu R. (2025). Artificial intelligence in clinical trials of lung cancer: Current and future prospects. Intell. Oncol..

[148] Wang Z., Xu W., Lei S., Lai Y., Zhang Y., Wang Y., Xiang Z., Fu X., Yang L. (2025). A computer-aided, carrier-free drug delivery system with enhanced cytotoxicity and biocompatibility: A universal model for multifunctional lung cancer therapy. Colloids Surf. B.

[149] Li J., Xiang L., Wang S., Zhang Y., Zhao S., Zhang D., Meng X., Zhang Y., Lin J. (2025). Targeting the crosstalk between glutamine metabolism and tumor immune microenvironment for lung cancer immunotherapy. Interdiscip. Med..

[150] Shoaib S., Islam N., Yusuf N. (2022). Phytocompounds from the medicinal and dietary plants: Multi-target agents for cervical cancer prevention and therapy. Curr. Med. Chem..

[151] Chen Y., Niu H., Fan L., Wen C., Wei X., Zhao Z., Surenjidiin S., Aruhan, Li M. (2024). Comprehensive analysis of *Perilla frutescens* (L.) Britt. along the value chain in northeast Inner Mongolia. Sci. Tradit. Chin. Med..

[152] Yi D., Wang Z., Peng M. (2025). Comprehensive review of perilla frutescens: Chemical composition, pharmacological mechanisms, and industrial applications in food and health products. Foods.

[153] Adam G., Robu S., Flutur M.-M., Cioanca O., Vasilache I.-A., Adam A.-M., Mircea C., Nechita A., Harabor V., Harabor A. (2023). Applications of Perilla frutescens extracts in clinical practice. Antioxidants.

[154] Youn I., Han S., Jung H.J., Noh S.G., Chung H.Y., Koo Y.K., Shin S., Seo E.K. (2023). Anti-inflammatory activity of the constituents from the leaves of Perilla frutescens var. acuta. Pharmaceuticals.

[155] Verspohl E.J., Fujii H., Homma K., Buchwald-Werner S. (2013). Testing of Perilla frutescens extract and Vicenin 2 for their antispasmodic effect. Phytomedicine.

[156] Zhong Y., Du Q., Wang Z., Zheng Q., Yang M., Hu P., Yang Q., Xu H., Wu Z., Huang X. (2024). Antidepressant effect of Perilla frutescens essential oil through monoamine neurotransmitters and BDNF/TrkB signal pathway. J. Ethnopharmacol..

[157] Guan H., Luo W., Bao B., Cao Y., Cheng F., Yu S., Fan Q., Zhang L., Wu Q., Shan M. (2022). A comprehensive review of rosmarinic acid: From phytochemistry to pharmacology and its new insight. Molecules.

[158] Fedenko V.S., Landi M., Shemet S.A. (2022). Metallophenolomics: A novel integrated approach to study complexation of plant phenolics with metal/metalloid ions. Int. J. Mol. Sci..

[159] Rao Q., Yu H., Li R., He B., Wang Y., Guo X., Zhao G., Wu F. (2024). Dihydroartemisinin inhibits angiogenesis in breast cancer via regulating VEGF and MMP-2/-9. Fund. Clin. Pharmacol..

[160] Menyailo M.E., Bokova U.A., Ivanyuk E.E., Khozyainova A.A., Denisov E.V. (2021). Metastasis prevention: Focus on metastatic circulating tumor cells. Mol. Diagn. Ther..

[161] Schuster E., Dashzeveg N.K., Tong F., Jia Y., El-Shennawy L., Zhang T., Hoffman A.D., Kitata R.B., Kibria G., Zhang Y. (2025). Computational ranking identifies Plexin-B2 in circulating tumor cell clustering with monocytes in breast cancer metastasis. Nat. Commun..

[162] Liu J., Wang L., Peng Y., Long S., Zeng H., Deng M., Xiang W., Liu B., Hu X., Liu X. (2025). A novel therapeutic strategy utilizing EpCAM aptamer-conjugated gemcitabine for targeting bladder cancer and cancer stem cells. Biomater. Sci..

[163] Song F., Wang C.-G., Wang T.-L., Tao Y.-C., Mao J.-Z., Hu C.-W., Zhang Y., Tang P.-J., Lu C.-L., Qing H.-L. (2024). Enhancement of gemcitabine sensitivity in intrahepatic cholangiocarcinoma through Saikosaponin-a mediated modulation of the p-AKT/BCL-6/ABCA1 axis. Phytomedicine.

[164] Salarvand A., Shanei A., Hejazi S.H., Abedi I., Kakhki N.A. (2025). In vitro Investigation of radiotherapy along with gemcitabine loaded PEG gold nanoparticles against MCF-7 breast cancer cells. Adv. Biomed. Res..

[165] Sun L., Shang H., Wu Y., Xin X. (2022). Hypericin-mediated photodynamic therapy enhances gemcitabine induced Capan-2 cell apoptosis via inhibiting NADPH level. J. Pharm. Pharmacol..

[166] Rajasekar S., Santhi K., Farheena M.I., Sampath K., Madhankumar R., Vetrivel C. (2023). Polydopamine coated molybdenum disulfide nanoflower as an efficient material for synergistic chemo-photothermal therapy. J. Clust. Sci..

[167] Jayan L.S., Rajan S.S., Mujahid S.M., Sharma S., Wani K.M. (2025). Capsaicin: An in-depth review of its chemical properties, health benefits, and challenges in food applications. Food Prod. Process Nutr..

[168] Mondal A., Banerjee S., Terang W., Bishayee A., Zhang J., Ren L., da Silva M.N., Bishayee A. (2024). Capsaicin: A chili pepper bioactive phytocompound with a potential role in suppressing cancer development and progression. Phytother. Res..

[169] Thongin S., Den-Udom T., Uppakara K., Sriwantana T., Sibmooh N., Laolob T., Boonthip C., Wichai U., Muta K., Ketsawatsomkron P. (2022). Beneficial effects of capsaicin and dihydrocapsaicin on endothelial inflammation, nitric oxide production and antioxidant activity. Biomed. Pharmacother..

[170] Vašek D., Fikarová N., Marková V.N., Honc O., Pacáková L., Porubská B., Somova V., Novotný J., Melkes B., Krulová M. (2024). Lipopolysaccharide pretreatment increases the sensitivity of the TRPV1 channel and promotes an anti-inflammatory phenotype of capsaicin-activated macrophages. J. Inflamm..

[171] Lima F.S., Rossi F.E., Gonçalves D.C., Clark N., Panissa V.L.G. (2022). Can capsaicinoids and their analogs affect energy expenditure and substrate oxidation during exercise? A systematic review with acute studies. Hum. Nutr. Metab..

[172] Szallasi A. (2022). Capsaicin for weight control: “exercise in a pill” (or just another fad)?. Pharmaceuticals.

[173] Chinreddy S.R., Mashozhera N.T., Rashrash B., Flores-Iga G., Nimmakayala P., Hankins G.R., Harris R.T., Reddy U.K. (2024). Unraveling TRPV1′s role in cancer: Expression, modulation, and therapeutic opportunities with capsaicin. Molecules.

[174] Zhang Y., Wu W., Li Y., Huang J., Chen J., Chen R., Mei Q., Huang Z., Sun J. (2025). Cyclometallated Ir (III) complexes exhibit antitumor activity through angiogenesis inhibition. Bioorg Chem..

[175] Wang Z., Yang L. (2020). GS-5734: A potentially approved drug by FDA against SARS-CoV-2. New J. Chem..

[176] Wang Z., Yang L., Song X.Q. (2022). Oral GS-441524 derivatives: Next-generation inhibitors of SARS-CoV-2 RNA-dependent RNA polymerase. Front. Immunol..

[177] Correia-da-Silva M., Sousa E., Pinto M.M. (2014). Emerging sulfated flavonoids and other polyphenols as drugs: Nature as an inspiration. Med. Res. Rev..

[178] Wang H., Abe I. (2025). Acetylation-deacetylation-driven natural product biosynthesis. ChemistryEurope.

[179] Mao X., Chen J., Yao Y., Liu D., Wang H., Chen Y. (2024). Progress in phosphorylation of natural products. Mol. Biol. Rep..

[180] Zhao M., Zhou H., Hao L., Chen H., Zhou X. (2021). Natural rosin modified carboxymethyl cellulose delivery system with lowered toxicity for long-term pest control. Carbohydr. Polym..

[181] Sung J.Y., Cheong J.H. (2026). Advancing genomics and integration of multi-omics for precision oncology using quantum machine learning. npj Digit. Med..

[182] Sahu R.C., Arora S., Kumar D., Agrawal A.K. (2026). Machine learning for predictive modeling in nanomedicine-based cancer drug delivery. Med. Res..

[183] Liu F., Beck S., Yang L., Luo H., Zhang K. (2026). Advancing AI for multi-omics and clinical data integration in basic and translational cancer research. Nat. Rev. Cancer.

